# A deep learning–enabled smart garment for accurate and versatile monitoring of sleep conditions in daily life

**DOI:** 10.1073/pnas.2420498122

**Published:** 2025-02-11

**Authors:** Chenyu Tang, Wentian Yi, Muzi Xu, Yuxuan Jin, Zibo Zhang, Xuhang Chen, Caizhi Liao, Mengtian Kang, Shuo Gao, Peter Smielewski, Luigi G. Occhipinti

**Affiliations:** ^a^Electrical Engineering Division, Department of Engineering, University of Cambridge, Cambridge CB3 0FA, United Kingdom; ^b^The Cavendish Laboratory, Department of Physics, University of Cambridge, Cambridge CB3 0FZ, United Kingdom; ^c^Department of Clinical Neurosciences, University of Cambridge, Cambridge CB2 0QQ, United Kingdom; ^d^Department of Ophthalmology, Beijing Tongren Hospital, Capital Medical University, Beijing 100005, China; ^e^School of Instrumentation and Optoelectronic Engineering, Beihang University, Beijing 100191, China

**Keywords:** sleep health monitoring, smart garment, e-textile sensor, deep learning, transfer learning

## Abstract

In wearable smart systems, continuous monitoring and accurate classification of different sleep-related conditions are critical for enhancing sleep quality and preventing sleep-related chronic conditions. However, the requirements for device–skin coupling quality in electrophysiological sleep monitoring systems hinder the comfort and reliability of night wearing. Here, we report a washable, skin-compatible smart garment sleep monitoring system that captures local skin strain signals under weak device–skin coupling conditions without positioning or skin preparation requirements. A printed textile-based strain sensor array responds to strain from 0.1 to 10% with a gauge factor as high as 100 and shows independence to extrinsic motion artifacts via strain-isolating printed pattern design. Through reversible starching treatment, ink penetration depth during direct printing on garments is controlled to achieve batch-to-batch performance variation <10%. Coupled with deep learning, explainable AI, and transfer learning data processing, the smart garment is capable of classifying six sleep states with an accuracy of 98.6%, maintaining excellent explainability (classification with low bias) and generalization (95% accuracy on new users with few-shot learning less than 15 samples per class) in practical applications, paving the way for next-generation daily sleep healthcare management.

## Significance

Sleep disorders are prevalent and affect the health and productivity of millions. Traditional sleep monitoring systems are complex and inconvenient for daily use. Our study introduces a smart garment that integrates a strain sensor array and deep learning to monitor sleep patterns accurately in a comfortable setting. This durable, artifacts-resilient, and positioning-free diagnostic e-textile can classify six healthy, subhealthy, and unhealthy sleep states with high accuracy and adaptability, making it a significant advancement over existing wearable technologies. With these unique features, the proposed solution marks a step forward in sleep medicine and consumer health, by providing a continuous, nonintrusive monitoring of sleep health, ultimately improving understanding and management of sleep disorders.

Sleep is a vital component of human health, occupying about one-third of daily life. However, over 60% of adults experience poor sleep quality, leading to significant personal and economic consequences, including the loss of 44 to 54 working days annually and a global GDP reduction estimated between 0.64% and 1.31% (1–3). Subhealthy and high-risk sleep patterns—such as mouth breathing, snoring, bruxism, and sleep apnea—are major contributors to poor sleep quality (4–7) and are linked to chronic diseases like cardiovascular disease, diabetes, and emotional disorders (8–11). Thus, effective monitoring and identification of these sleep states are crucial for modern health management.

Traditional sleep monitoring relies on polysomnography, which is considered the gold standard for diagnosing sleep disorders due to its ability to capture comprehensive physiological signals such as electroencephalograms (EEG), electrooculograms (EOG), and electromyograms (EMG) (12). However, its complexity, cost, and invasive nature make it impractical for home or long-term use. To address this, portable alternatives like Home Sleep Apnea Testing (HSAT) systems have been developed, guided by the American Academy of Sleep Medicine (AASM) classification. These systems are categorized based on the number and type of signals they collect, with higher-type systems incorporating multiple modalities (e.g., EEG and EOG in Type II) and lower types (e.g., Type III and IV) focusing on simplified monitoring of parameters like airflow, oxygen saturation, and heart rate. Despite these advancements, HSAT systems are often condition-specific, primarily targeting sleep apnea, and may not address broader sleep monitoring needs. To enhance usability and accessibility, smart wearable devices have emerged as promising alternatives (13–15), integrating sensors such as photoplethysmography (PPG) within watches or wristbands (16–18). These devices, while user-friendly, often lack the ability to capture the rich physiological data required for thorough analysis of various sleep states (19). More sophisticated designs incorporating physical sensors—such as humidity, mechanical, and acoustic sensors—have been proposed to bridge this gap (20–24). Although these systems provide broader physiological insights, they typically require multiple integrated sensors, increasing bulkiness, energy consumption, and compromising comfort for long-term use (20, 22, 23). Additionally, attempts to integrate noninvasive electrophysiological sensors, such as EEG, EOG, and EMG, into wearable devices on facial or ear areas have shown promise in improving data richness and comfort (25–27). However, these systems still face challenges with motion artifacts and inherent noise, which can limit their reliability and accuracy, especially in real-world applications (28, 29). Consequently, there remains a need for versatile, user-friendly technology capable of accurately and comprehensively monitoring diverse sleep conditions in daily environments.

**Fig. 1.**
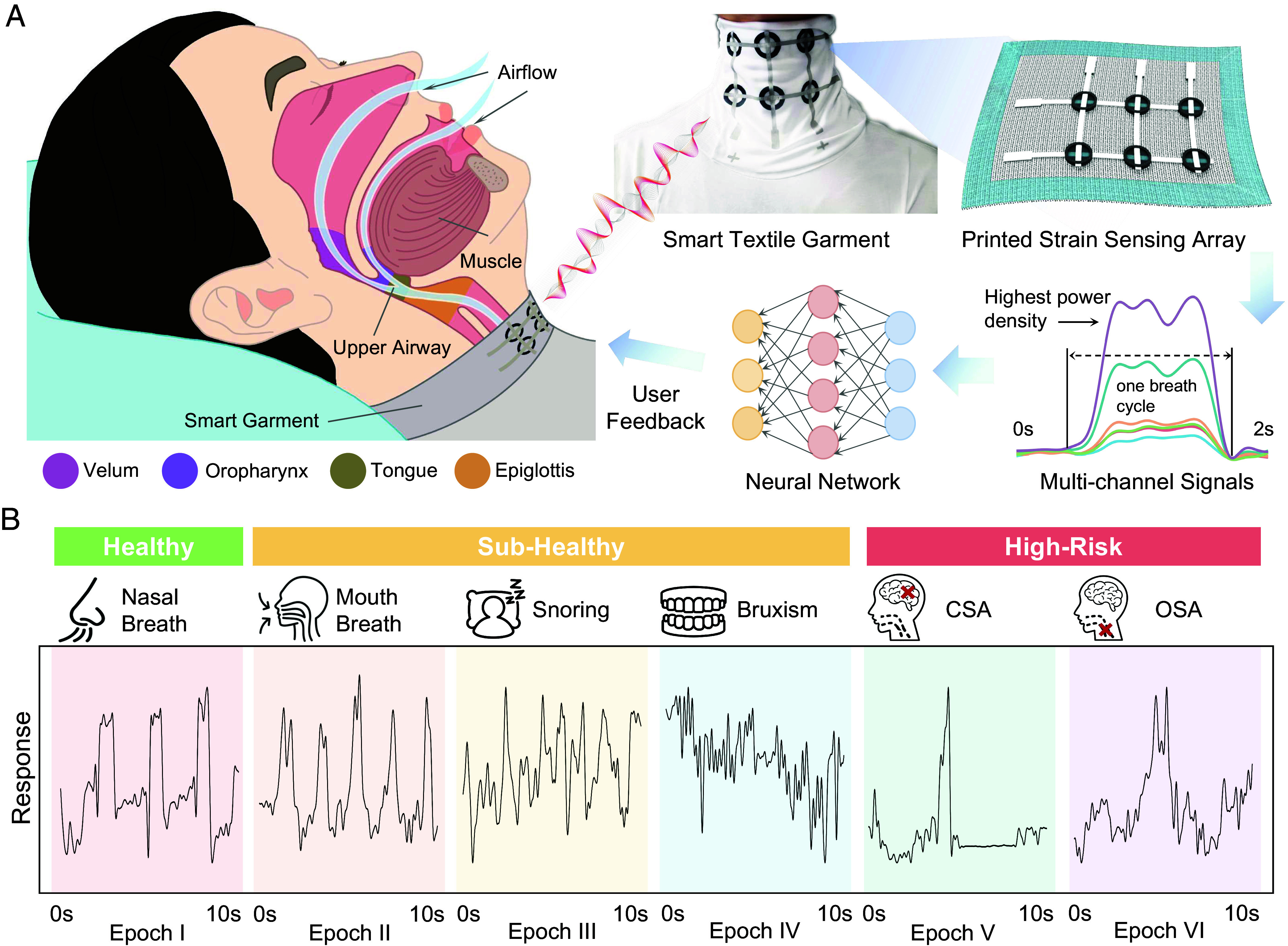
Overview of the smart garment system for versatile sleep behavior monitoring. (*A*) The monitoring of sleep behavior begins by detecting subtle vibrations at the extrinsic laryngeal muscle, which are induced by physiological vibrations emanating from various anatomical locations such as the velum, oropharynx, tongue, and epiglottis. These vibrations are then captured by a six-channel strain sensor array printed onto the collar of a garment. The signals from the channel with the strongest response are processed by a deep learning neural network, SleepNet, which is designed for recognizing and analyzing sleep patterns. (*B*) Visualization of the signals of six different sleep patterns (channel with the highest power density).

Here, we developed a smart garment that balances the trade-off between versatility, comfort, and accuracy for monitoring sleep health in daily environments. The smart garment is integrated with an ultrasensitive sensor array directly printed on the collar, capable of identifying multiple sleep patterns. Direct printing on textile substrates enables the integration of multifunctional electronic elements directly onto cloths with scalability and design flexibility. The developed smart garment is able to collect mixed-mode signals generated by vibrations from various sleep activities such as breathing, snoring, teeth grinding, and sleep apnea, which are transmitted to the extrinsic laryngeal muscles from multiple anatomical locations including the velum, oropharynx, tongue, and epiglottis (Fig. 1*A*). Utilizing a multichannel graphene textile strain sensor array, screen printed at its collar based on principles of ordered cracks and selective starching treatments, the smart garment leverages ultrahigh sensitivity (gauge factor >100), scalability (±20% conductivity fluctuation), and durability (stable over 10,000 cycles of stretching tests) to continuously monitor subtle vibrations of the extrinsic laryngeal muscles while ensuring user comfort. Additionally, our designed strain isolation treatment allows to adjust the substrate’s rigidity, isolating strain artifacts caused by nocturnal turnings and other motions outside the sensing area, thereby significantly reducing the magnitude and drift of motion artifacts. Additionally, due to its multichannel design, the smart garment can be easily used by wearers in a positioning-free manner in real-world application scenarios. The designed deep learning model, SleepNet, uses the captured signals to accurately identify six sleep states ranging from healthy to subhealthy to high-risk, including nasal breath, mouth breath, snoring, bruxism, central sleep apnea (CSA), and obstructive sleep apnea (OSA), achieving an accuracy of 98.6% along with decent inference speed (Fig. 1*B*). Explainable AI visualizations confirm that the model comprehensively understands the sleep patterns, avoiding biases toward noisy regions, thus demonstrating its robustness. Moreover, transfer learning tests show that after few-shot learning (with only 15 samples per class), the model can achieve up to 95% classification accuracy on new users, showcasing the system’s powerful generalization capabilities. In Table 1, we have summarized the functions and features of our system compared with other wearable sleep monitoring systems in the literature, showing that, to the best of our knowledge, the proposed system consistently outperforms state-of-the-art solutions in functionality performance, reliability, versatility, and user-convenience. Given its user-friendly design, high accuracy, and personalization features, the proposed technology favors the adoption of this smart garment for daily use, empowering individuals to better understand and manage their sleep health and promoting well-being for a wide range of users.

**Table 1.** Comparison of the proposed smart garment’s features with state-of-the-art wearable sleep monitoring systems

	This work	Shen et al. (16)	Yue et al. (19)	Sun et al. (21)	Tarim et al. (22)	O’Hare et al. (23)	Kwon et al. (24)
Form factor	Smart textile garment	Smart bracelet	Philtrum and neck e-skin	Philtrum and chest patch	Wearable platform	Masseter EMG patch	Smart face patch
Sensors	6-channel strain sensor	1 PPG sensor	1 pressure, 1 humidity, 1 temperature sensor	1 pressure and 1 humidity sensor	1 IMU and 1 temperature sensor	1 bipolar surface EMG	2-channel EEG, 2-channel EOG, 1-channel EMG
Functions	Mouth breath detection	Sleep apnea detection	Sleep stage classification	Sleep apnea detection	Breath patterns detection	Bruxism detection	Sleep apnea detection
	Snoring detection						Sleep quality assessment
	Bruxism detection						
	Sleep apnea detection						
Scalability	Good	Good	Bad	Bad	Good	Good	Good
Robustness	Good	Medium	Bad	Bad	Medium	Medium	Medium
Durability	Good	Good	Good	Good	Good	Bad	Good
Washability	√	×	×	×	×	×	√
Breathability	Good	Good	Medium	Bad	Good	Bad	Medium
No precise positioning needed	√	√	×	×	×	×	×
Overall accuracy	98.6%	81.82%	N/A	N/A	96%	82.8%	88.5%
Generalization ability	Good	Good	Good	Good	Good	Medium	Medium
Explainability	Good	Good	Good	Good	Good	Bad	Bad

Note: N/A indicates that this feature was not tested in the literature and does not necessarily imply that the method lacks this capability in principle.

## Results

### Printed Strain Sensor Array on Garments.

**Fig. 2.**
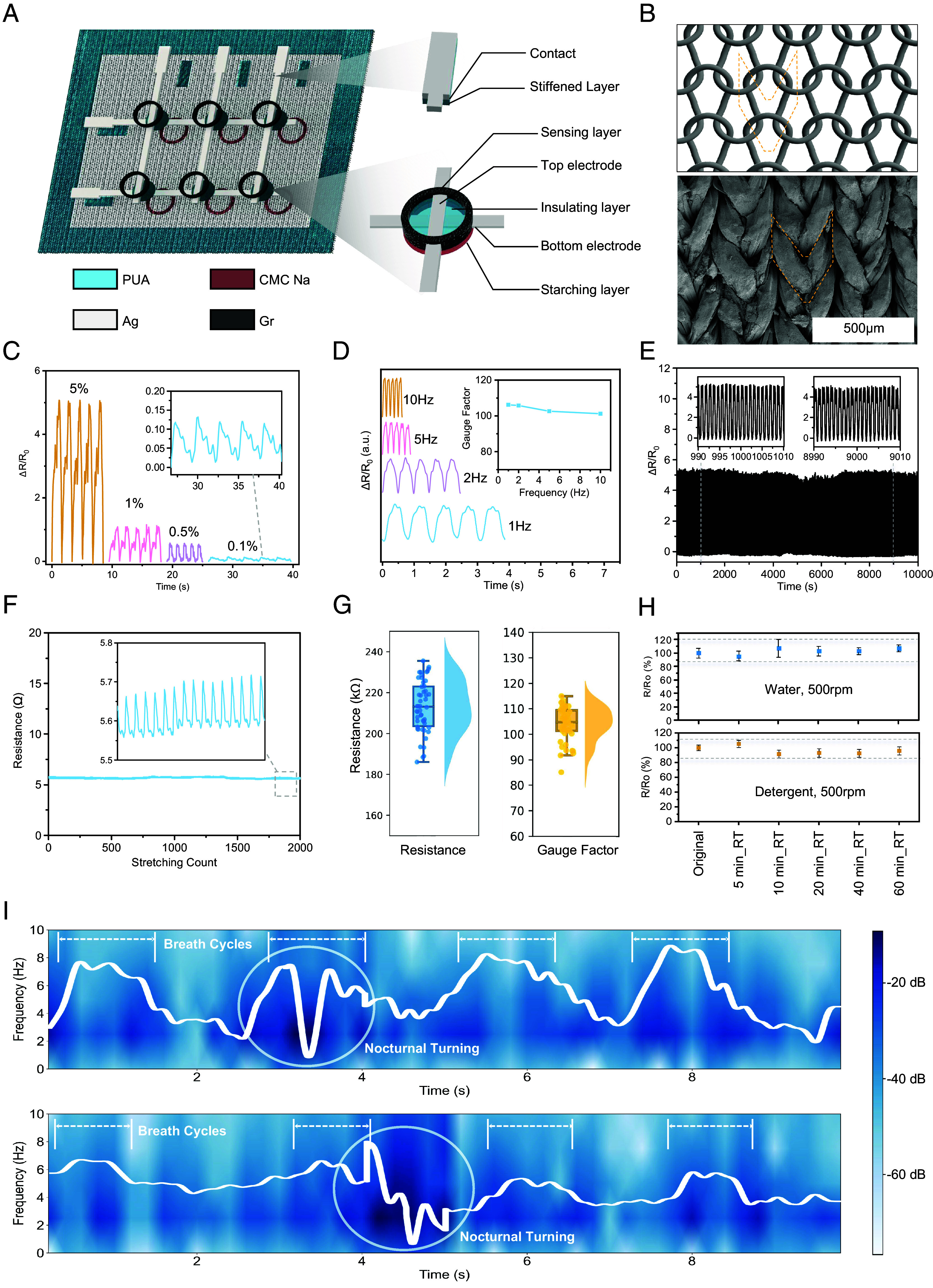
Characterization of the device. (*A*) Schematic of the strain sensor array, including the elastic spandex textile substrate, starching layer, crossbar electrodes, and circular sensing layer. (*B*) Schematic (*Top*) and SEM image (*Bottom*) of the ordered cracks formed around the textile structural units after graphene printing. The structural units are labeled with yellow dashed lines. (*C*) Resistance response to cyclic tensile strains of 5%, 1%, 0.5%, and 0.1%. *Inset* shows a zoomed-in view of the response at 0.1%. (*D*) Dynamic response test under uniaxial cyclic tensile at 1% strain with different frequencies: 10 Hz, 5 Hz, 2 Hz, and 1 Hz. *Inset* shows the gauge factor at each frequency. (*E*) Durability test of graphene strain sensors under 10,000 cycles of 1% strain. (*F*) Strain response of stretchable silver electrodes under 1% strain. (*G*) Raincloud plot of resistance and gauge factor measurements for 50 strain sensor units. The density plots, dot plots, and box plots show the distribution, median, and variability of resistance and gauge factor, respectively. (*H*) Washability test under room temperature (RT) and 500 rpm stirring with a magnetic stir bar. Gray dashed lines show the maximum and minimum values measured during the experiment. (*I*) The nasal breath signals during nocturnal turning collected from the device with (*Upper*) and without (*Lower*) strain isolation treatment.

Fig. 2*A* illustrates the multilayered structure of the strain-sensing array screen-printed on a high-neck top made of elastic knitted fabric. In the multilayer screen printing process, printing quality can be easily adapted to different textile substrates by tuning the printing parameters and ink properties. To ensure stable performance, we introduced two different treatments in the fabrication process: starching treatment using sodium carboxymethyl cellulose (CMC Na), for the sensing area, and strain isolation treatment using polyurethane acrylate (PUA), for the surrounding regions. Both cellulose derivatives and acrylates are common starching agents in garment printing industry (30). CMC Na is a water-soluble polymer derived from cellulose known for its film-forming ability, which provides a smooth and uniform surface (31). CMC Na also improves the adhesion of the printed graphene ink to the substrate, reducing the likelihood of delamination under mechanical stress. The PUA provides high stiffness and excellent adhesion by forming a robust, cross-linked network upon UV exposure (32). Introducing a rigid PUA layer in the textile strain sensor array modifies the rigidity of selected area on the textile, redistributing strain caused by body movements during sleep (*SI Appendix*, Fig. S1). The isolated area remains inert to large-scale uniaxial stretching, ensuring that local strain is accurately measured without interference, as shown in *SI Appendix*, Fig. S3. The UV-curable nature also prevents clogging of the screen during the printing process, extending the lifespan of the printing screen and maintaining consistent printing quality.

After the starching and strain isolation treatment, the crossbar silver electrodes, separated by an insulating layer, are printed, followed by the graphene sensing layer on the surface, which comprises exfoliated graphene flakes bound with ethyl cellulose (EC). The graphene flakes production process is tuned to obtain flakes under 1 μm lateral dimension to ensure stable dispersion in the graphene ink formulation and avoid mesh clogging (*SI Appendix*, Fig. S4). Controlling the formation of ordered cracks in the graphene layer is critical for fabricating crack-based strain sensors with repeatable performance (33). During the screen-printing process, the ink is squeezed through the mesh by the squeegee, resulting in a patterned thin film onto the textile substrate. The stress concentration at the boundaries of the textile structural units induces the formation of regular cracks (Fig. 2*B*). This process does not require complex prestretching or pretreatment steps, making it compatible with conventional printing processes adopted by the garment manufacturing industry (34). A known issue in textile printing processes is related to capillary forces causing the ink to spread, while air pockets block ink deposition, leading to variability and poor printing quality. Additionally, in this case, excessive penetration of graphene ink into the textile can create a graphene/textile composite which is insensitive to strain and acts as an extra conductive pathway to the surface cracking layer (*SI Appendix*, Fig. S5). To overcome these limitations and inhibit ink penetration and air trapping during the printing process, another starching treatment with CMC Na was used, which creates a controlled surface for ink deposition, preventing deep penetration and ensuring the graphene forms a brittle surface layer that cracks under strain. As shown in *SI Appendix*, Fig. S7, the penetration depth of graphene ink in starched textile is significantly lower compared to untreated textile. The fabrication process is illustrated in *SI Appendix*, Fig. S8. It is scalable and compatible with industrial textile printing processes, making it suitable for mass production of smart garments (35).

The graphene/EC coating with ordered cracks shows a reliable linear response to small uniaxial strains from 0.1 to 5% with a gauge factor over 100 (Fig. 2*C*). Moreover, the strain sensors demonstrate a rapid response to straining cycles with frequency ranging from 1 Hz to 10 Hz (Fig. 2*D*), enabling the real-time monitoring of fast and subtle vibrations produced by the throat during sleep. The durability of the graphene sensor was tested through tensile tests at 1% uniaxial strain and 1 Hz frequency, showing consistent performance (Fig. 2*E*). In contrast to the high response of the strain-sensing layer, the stretchable silver electrodes exhibit high conductivity with negligible strain response, ensuring stable electrical connections (Fig. 2*F*). To ensure consistency and reliability, we studied the performance distribution of two-terminal resistance and gauge factor across 50 strain sensor units. A resistance variation of less than 12.7% and a gauge factor variation of less than 9.2% were achieved by controlling printing conditions and applying starching treatments, as shown in Fig. 2*G*. Moreover, as observed from Fig. 2*I*, compared to the device without PUA treatment, the device with treatment shows significant mitigation on the amplitude and drift of artifact signal during nocturnal turnings, which validates the effectiveness of strain isolation treatment.

The washability of the sensors was tested by immersing the devices in water under 500 rpm stirring for 5 to 60 min, drying at room temperature (36). The performance degradation after washing was less than 10%. Similar results were observed when detergent was added (Fig. 2*H*). Additionally, the smart garment substrate maintains excellent breathability, even after treatment and integration of the sensing devices, with moisture vapor transmission rate values over 10 times higher than Tegaderm (37), a commonly used medical dressing substrate produced by 3M (*SI Appendix*, Fig. S9). Furthermore, the strain sensor array results in no skin irritation or other side effects after 8 h of wear, as the typical duration of an overnight sleep (*SI Appendix*, Fig. S10). Artificial sweat was used to test the resistance of the smart garment against prolonged use in sweat environment (ISO 3160-2 standard), resulting in no signs of degradation of its electrical resistance even after 2 h of exposure to both normal (1 h at 0.32 mg/cm^2^/min) and high (1 h at 2.7 mg/cm^2^/min) sweat rate (*SI Appendix*, Fig. S11).

### Positioning-Free Monitoring of Sleep Conditions with the Array.

**Fig. 3.**
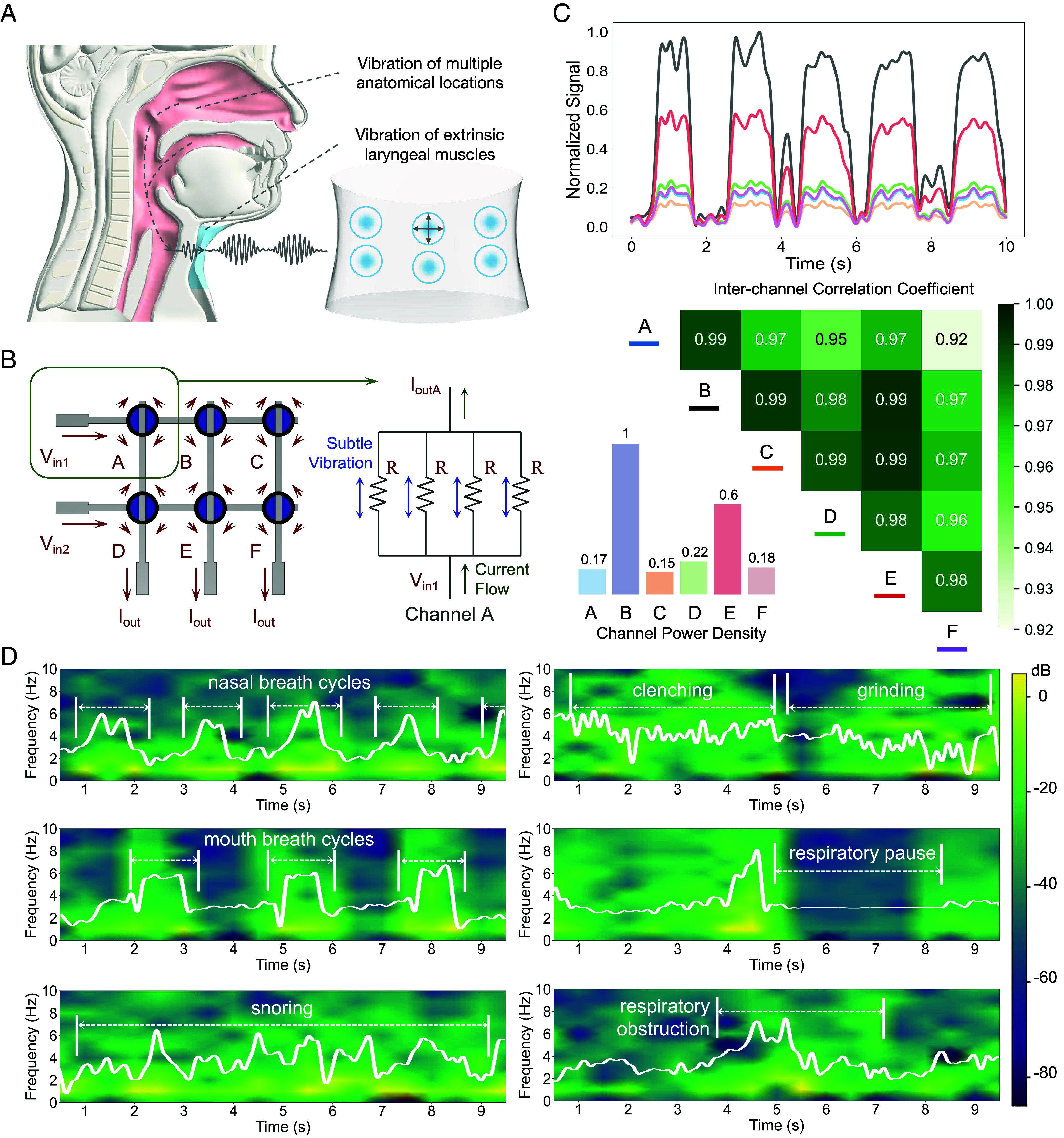
Performance of the strain sensor array in acquiring sleep conditions–related signals. (*A*) Illustration of how sleep behavior generates extrinsic laryngeal vibrations. (*B*) Equivalent circuit of the strain sensor array. (*C*) Multichannel signal and channel correlation visualization. (*D*) Time–frequency spectrogram of the channel signal with the strongest response.

We employed a six-channel strain sensor array integrated into the collars of textile garments, positioned around the participants’ necks. This setup was designed to collect minute vibrational signals from the extrinsic laryngeal muscles, which vary according to different sleep conditions (Fig. 3*A*). Six channels provide sufficient spatial resolution to capture detailed vibrational patterns associated with various sleep conditions. A larger number of sensors would increase complexity and data processing demands with no significant gain in accuracy, while a smaller number might fail to capture critical signals (*SI Appendix*, Fig. S21). This configuration allows for reliable detection of sleep states across a diverse range of users. Utilizing a multiplexer, we could read the responses from a circular six-channel piezoresistive strain sensor arranged in a crossbar structure for further analysis. In our design, each circular sensing channel can be equivalently viewed as a parallel connection of four quarter-ring variable resistors, as shown in Fig. 3*B*, when we reflect the resistance values of the vertical and horizontal lines of the sensors. This design provides two-dimensional sensitivity to both horizontal and vertical strain. The sensor area was optimized by arranging the channels to maximize coverage around the neck, ensuring that at least one sensor captures the strongest response, regardless of garment positioning. This layout minimizes the need for precise positioning and enhances usability, allowing users to wear the garment comfortably without worrying about keeping the sensors aligned with the breathing-related sensitive areas of the neck region. Fig. 3*C* illustrates the response signals during a 10-s sample of nasal breathing, captured by the six channels at standard wearing positions. Correlation analysis revealed that although the intensity of the strain responses varied across different channel locations covering the throat area, the signal characteristics were highly correlated (Pearson correlation coefficients greater than 0.9 between any two channels). This pattern persisted even when the sensor array was worn askew, not in the standard position (*SI Appendix*, Fig. S12). These findings demonstrate that our design, which utilizes a relatively large coverage area of the strain sensor array, essentially ensures that the region with the strongest response falls within the device’s sensitive area. Consequently, our sleep conditions monitoring system does not require precise positioning, implying high resilience to positional variances, which ensures the practicality of this monitoring system in real-world application scenarios. Furthermore, due to the high correlation between channels, subsequent neural network-based pattern recognition needs only to consider the channel with the strongest response as representative for signal processing and extraction of the relevant features, which improves the network’s inference efficiency while ensuring accuracy.

To ensure effectiveness of the proposed approach, we built a comprehensive dataset encompassing six distinct classes of sleep patterns: nasal breathing (normal healthy condition), mouth breathing, snoring, bruxism, CSA, and OSA. The dataset was collected from seven healthy subjects and spans a health spectrum from healthy and subhealthy to high-risk categories. Therefore, for the three conditions—bruxism, OSA, and CSA—which are rare among healthy individuals, the data were augmented through simulated conditions under the guidance of medical experts. In contrast, data for mouth breathing, nasal breathing, and snoring were collected from actual sleep sessions in different subjects. Detailed protocols for data collection are described in the *Materials and Methods* section. As visualized in Fig. 3*D*, the temporal and spectral characteristics of these sleep conditions were meticulously analyzed focusing on the signals emanating from the channel exhibiting the strongest response. We observed that the effective vibrational signals originating from the extrinsic laryngeal muscles are predominantly found within the low-frequency domain, specifically below 10 Hz. This frequency band captures the physiological nuances of each sleep pattern, enabling a precise delineation of the conditions. Nasal and mouth breathing exhibit fundamental differences in their spectral signatures, reflecting variations in airflow mechanics and potential diagnostic markers for respiratory efficiency. The irregular and prominent vibrational patterns associated with snoring suggest the presence of disrupted airflow dynamics and serve as indicators of upper airway resistance. Notably, the episodic high-amplitude signals of bruxism provide clear evidence of nocturnal teeth grinding, which could be associated with high stress levels or sleep disturbances. The absence of vibrational activity during respiratory pauses in CSA and the erratic signal fluctuations indicative of breathing efforts against obstruction in OSA are consistent with the clinical understanding of these two conditions. More visualization of the samples is shown in *SI Appendix*, Figs. S13–S18.

### Sleep Conditions Recognition with a Deep Learning Model.

The preprocessing involves labeling (see detailed protocols in *SI Appendix*, Note S2), segmentation (10 s signals were segmented into one sample, and 50% overlapping was introduced for data augmentation), and Z-score normalization (38). The 10-s interval for segmentation was chosen to balance capturing essential temporal features of sleep events with computational efficiency. This duration effectively captures the necessary contextual information for identifying patterns like breathing cycles and snoring while avoiding the noise and inefficiency of longer segments, thus ensuring accurate and real-time processing. To enhance model robustness and data augmentation, a 50% overlapping across adjacent time series was used, which increases the number of training samples and improves the model’s ability to generalize across varied sleep patterns. Z-score normalization was applied to standardize the data, eliminating biases caused by amplitude variations and ensuring consistent input scales for accurate pattern recognition.

**Fig. 4.**
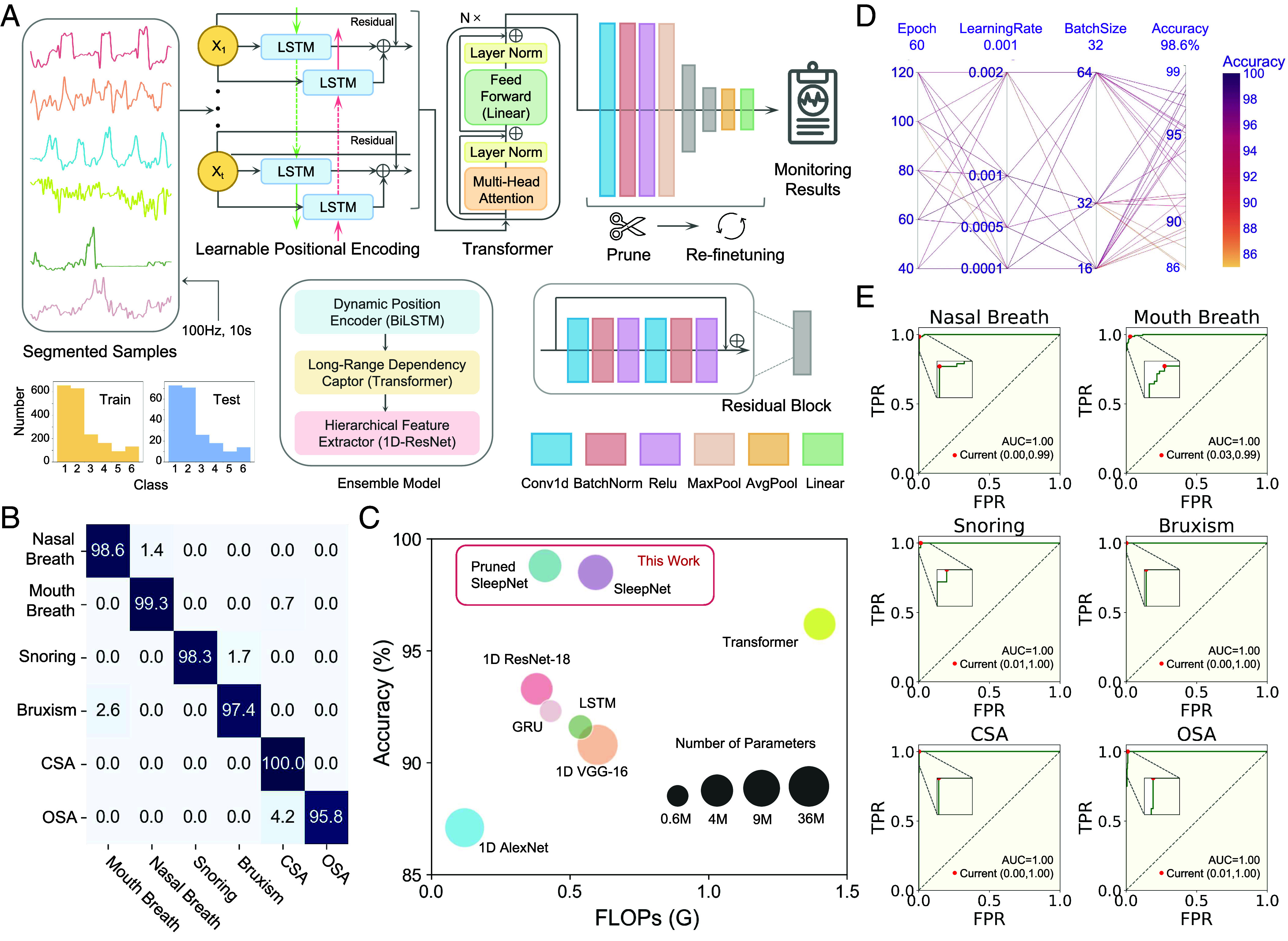
The sleep conditions recognition model. (*A*) Pipeline of the SleepNet for sleep pattern recognition. (*B*) Confusion matrix showing the classification results for six sleep patterns. (*C*) Comparison of model efficiency (measured in FLOPs), accuracy, and number of parameters with state-of-the-art neural network backbones. (*D*) Process visualization of random hyperparameter optimization. (*E*) Six receiver operating characteristic (ROC) curves and area under the curve (AUC) values of classification by the pruned SleepNet.

After that, signals from the channel with the strongest response were fed into our specially designed deep learning model (SleepNet) for sleep conditions recognition (Fig. 4*A*). The SleepNet is composed of three core components: First, the learnable positional encoder adopts a residual bidirectional LSTM (BiLSTM) framework to understand the sequential nature of the input data. This approach surpasses traditional sinusoidal positional encoding by dynamically learning positional information, which is particularly advantageous in sleep pattern analysis where the temporal relationship between events can signify different breath cycles or disturbances (39). Second, the multihead self-attention module, derived from Transformer architectures, enables nuanced discrimination of significance across the data sequence. By effectively utilizing long-range dependencies, the model ensures a comprehensive understanding of the sequential data, reflecting the true complexity of sleep behaviors over time (40). Finally, the one-dimensional Residual Network (ResNet) layers function as a hierarchical feature extractor. With their ability to skip connections, they prevent the vanishing gradient problem and enhance the flow of information, thus allowing the model to learn more complex patterns effectively. The 1D convolution within these layers ensures that the model is tailored to handle time-series data, offering a nuanced analysis of temporal patterns during sleep (41).

Fig. 4*B* displays the confusion matrix for the model’s classification of sleep patterns. The model achieved impressive classification results: The accuracy for each category was above 95%, with an overall accuracy of 98.6%. In the comparative experiments shown in Fig. 4*C*, SleepNet surpassed the performance of its individual component architectures, such as the Transformer and 1D ResNet, as well as other state-of-the-art models in terms of accuracy. Additionally, it is noteworthy that, after pruning the least important 50% of the nodes in the 1D-ResNet module of the model and retraining to obtain a pruned SleepNet, not only did the number of floating-point operations per second (FLOPS)—a critical indicator of model inference speed and energy efficiency—decrease by 30%, but there was also a slight increase in accuracy. This can be attributed to the phenomenon known as “pruning-induced efficiency,” where removing redundant or less important nodes can lead to a more streamlined and efficient network. The retraining phase helps the model to reallocate its resources toward the most salient features, potentially improving generalization and thus accuracy (42). Overall, the pruned SleepNet achieved less than 0.5 GFLOPs and fewer than 5 million parameters, highlighting its capability for deployment on edge computing devices. This efficiency allows the model to run effectively on low-power, resource-constrained hardware, making it suitable for real-time sleep monitoring in wearable devices without compromising performance.

Fig. 4*D* illustrates the process of hyperparameter optimization for the model. It can be observed that in the majority of hyperparameter combinations, the model exhibits high accuracy (greater than 90%). This indicates that the model’s performance is not overly sensitive to the specific values of its hyperparameters, demonstrating the model’s robustness. Moreover, such robustness might also imply that the fundamental features learned by the model are strong predictors of sleep patterns, allowing for decent performance despite variations in model configuration. Fig. 4*E* shows the ROC curves for the model’s classification of each sleep pattern type. The AUC values are almost equal to 1 for each classification task, indicating that the model is effective and has achieved satisfactory classification performance.

**Fig. 5.**
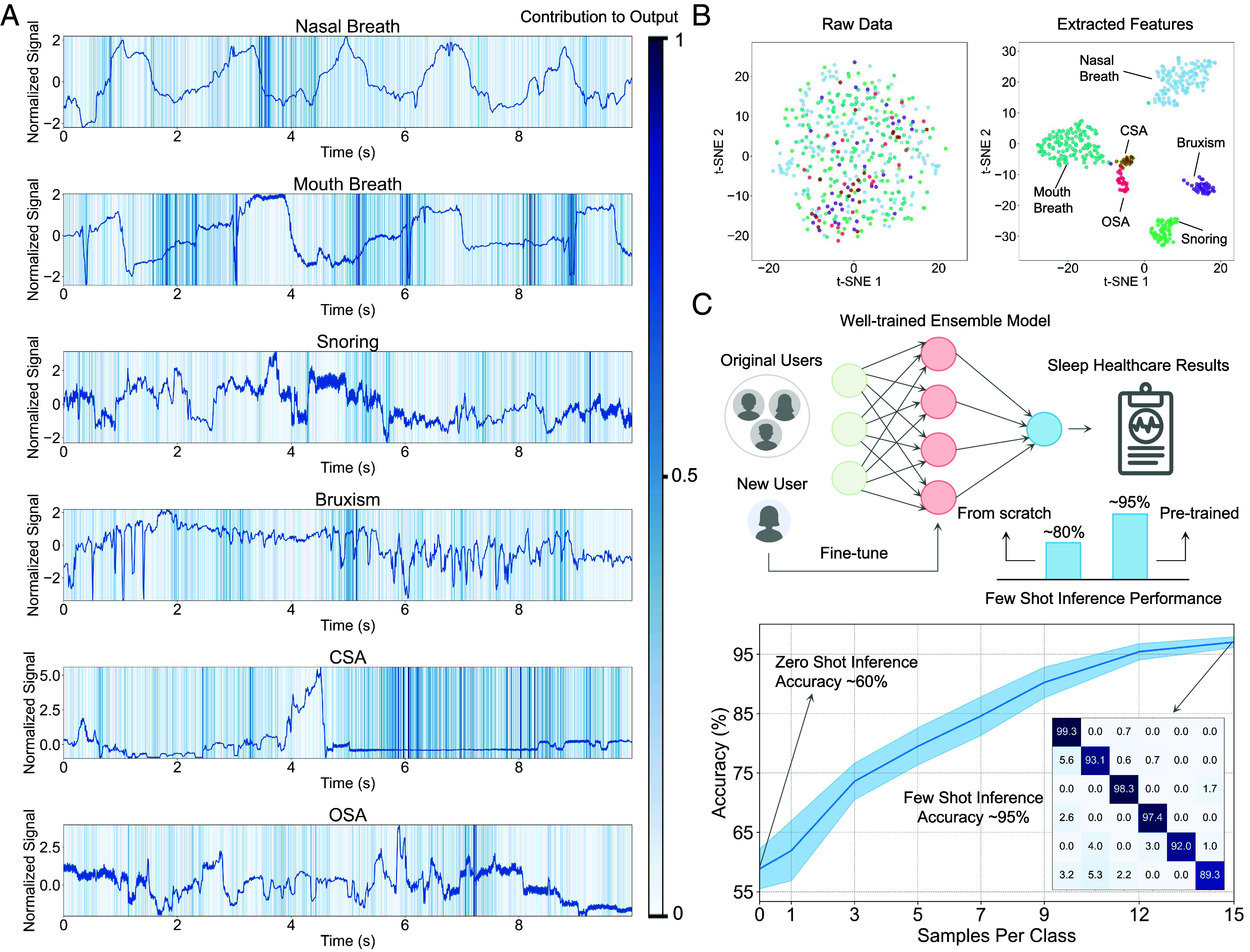
Explainability and generalizability analysis. (*A*) SmoothGrad displays the distribution of contributions that signals make to the model’s classification output. (*B*) T-distributed stochastic neighbor embedding (t-SNE) visualizations comparing the distribution of raw data to features extracted from the model. (*C*) Flowchart and results depicting the model’s generalization process.

SmoothGrad visualizations in Fig. 5*A* provide a clear depiction of how different segments of the signal contribute to the model’s classification decisions (43). The shading intensity represents the degree to which each point in the time series influences the output, with darker shades indicating higher importance. For instance, the “Nasal Breath” and “Mouth Breath” classifications exhibit consistent contribution patterns throughout the breathing cycle, reflecting the model’s reliance on rhythmic features for these classes. Conversely, “Snoring” shows a more variable contribution pattern, which likely corresponds to the erratic nature of snoring events. The “Bruxism” class demonstrates pronounced contributions at peaks, which may correspond to teeth-grinding instances. In the cases of “CSA” and “OSA,” the model identifies critical contributions at pause cycle and obstruction cycle. This distribution of contributions is consistent with established physiological patterns, which means that the model gives appropriate weight to relevant features across different classes, reflecting a balanced understanding of the data rather than an overreliance on certain input aspects that could lead to skewed predictions (e.g., noise). Furthermore, this underscores the model’s capacity to not only recognize but also assign appropriate significance to the distinct temporal features within the complex landscape of sleep-related signals, enhancing the interpretability of its predictions. Fig. 5*B* compares the distribution of raw data with the features extracted by the model in the t-SNE plane, illustrating the model’s ability to discern and delineate the complex structure within the data. The t-SNE visualization of extracted features shows distinct clusters corresponding to different sleep patterns, which are not easy discernible in the raw data. This indicates the effectiveness of the feature extraction in capturing the patterns and underlying relationships necessary for accurate classification.

To assess the model’s generalization ability, we conducted transfer learning tests, applying the model trained on five participants to the dataset of two new participants. As shown in Fig. 5*C*, with only 15 samples per category for few-shot learning, the model achieved an accuracy of up to 95% on the new subject. In contrast, training only on the new participant’s dataset without transfer yielded a few-shot learning accuracy of merely 80%. This reflects the model’s ability to adapt and maintain performance across different individuals, showcasing its robust transferability and capacity to leverage previously learned patterns to quickly adapt to new, unseen data.

## Discussion

Decoding human sleep patterns is important yet complex. Despite the promising development of wearable devices for sleep health monitoring in recent years, creating a system that combines versatility, comfort, and accuracy remains a significant challenge, hindering adoption. In this work, we have designed a smart garment integrated with a six-channel strain sensor array. This ultrasensitive strain sensor array, characterized by excellent robustness and durability, can collect subtle vibrations from the extrinsic laryngeal muscles associated with various sleep patterns, and its multichannel design eliminates the need for positioning due to its spatial resolution. Specifically, the strain isolation treatment mitigates strain artifacts caused by nocturnal movements and other motions outside the sensing area, which ensures the long-term reliability and robustness of the smart garment during overnight use. Despite utilizing only a single modality of strain response signals, our smart garment, equipped with a customized deep learning neural network, can comprehensively analyze and recognize subtle vibrations originating from various physiological sites and transmitted to the extrinsic laryngeal muscles. It accurately classifies six sleep patterns: nasal breath, mouth breath, snoring, bruxism, CSA, and OSA. Additionally, it can efficiently and effectively adapt to new users, maintaining high accuracy in its classifications. We believe that our smart garment offers a promising solution for versatile sleep monitoring in wearable devices, suitable for broad consumer electronics market to provide ongoing sleep monitoring for general users.

An important direction for future work involves conducting broader user studies with the smart garment across diverse populations. While our study demonstrated the system’s effectiveness among healthy users of varying body types and gender within a young demographic, it would be valuable to investigate whether the microvibration patterns of the extrinsic laryngeal muscles during different sleep states exhibit consistent characteristics across a wider range of age groups, including both healthy individuals and patient populations. Such exploration would not only enhance our understanding of the system’s applicability but also provide deeper insights into the fundamental principles of sleep. If current strain signals prove insufficient to develop a universal model for more diverse groups, integrating demographic characteristics (e.g., age, gender, body type) and prior information (e.g., key daytime activities) to create personalized models would be a meaningful approach (14). Additionally, while this study has quantified the feasibility of the system for real-time monitoring in terms of FLOPs and parameter size, future work should focus on integrating the smart garment into a truly wireless edge-computing system addressing low energy consumption and latency requirements for application in real-world settings. With these further developments, the proposed smart garment has the potential to become a benchmark in sleep monitoring technology.

## Materials and Methods

### Materials.

TIMREX KS 25 Graphite (particle size of 25 μm) was sourced from IMERYS. Stretchable conductive silver ink was obtained from Dycotec Materials Ltd. Ethyl cellulose and sodium carboxymethyl cellulose were purchased from SIGMA-ALDRICH. Flexible UV Resin Clear was acquired from Photocentric Ltd. The textile substrate, composed of 95% Polyester and 5% spandex, was procured from Jelly Fabrics Ltd.

### Ink Formulation.

The graphene ink for screen printing was prepared following a reported method. Briefly, 100 g of graphite powder and 2 g of EC were mixed in 1 L of isopropyl alcohol (IPA) and stirred at 3,000 rpm for 30 min. The mixture was then added into a high-pressure homogenizer (PSI-40) at 2,000 bar pressure for 50 cycles to obtain graphene dispersion. The graphene dispersion is centrifuged at 5,000 g for 30 min to remove unexfoliated graphite. To prepare the CMC Na starching solution, CMC Na was dissolved in water at 20% wt. concentration.

### Fabrication of Textile Strain Sensor Arrays.

The textile substrate was washed with detergent, thoroughly dried, and then treated with UV-ozone for 5 min to clean the surface. Screen printing was performed using a 165T polyester silk screen on a semiautomatic printer (Kippax & Sons Ltd.) set with a squeegee angle of 45 degrees, a spacer of 2 mm, a coating speed of 10 mm/s, and a printing speed of 40 mm/s. Printing pressure was pneumatically controlled, with higher pressure applied for the viscous starching agent, and moderate pressure for the thinner graphene ink silver ink to reduce penetration. After each printing pass, the textile was blown to dry. After printing, the sensor was washed with water to remove CMC Na and dried at 80 °C overnight. A biaxial strain of around 10% was then applied to induce the formation of ordered cracks.

### Characterization of Structure and Performance.

The size distribution of graphene flakes was analyzed using a Bruker Icon atomic force microscope in an area approximately 20 μm × 20 μm. Scanning electron microscopy (SEM) images were taken with a Magellan 400, after sputtering the textile samples with a 5 nm layer of gold to enhance conductivity. Optical images were captured using an Olympus microscope.

### Tensile Tests.

Tensile properties of the textile strain sensors were evaluated using a Deben Microtest 200N Tensile Stage and an INSTRON universal testing system. Electrical signals were recorded concurrently with a potentiostat (EmStat4X, PalmSens) and a multiplexer (MUX, PalmSens). Copper tape was crimped onto the contact pads of the samples, supplemented with a small amount of silver paste to improve electrical contact.

### Experimental Setup of Data Acquisition.

Our strain sensors were screen printed onto the collars of garments, equipped with copper strips at the crossbar electrodes. For data acquisition, we employed a potentiostat (EmStat4X, PalmSens) and a multiplexer (MUX8-R2, PalmSens) as our primary readout modules. These modules consistently supplied a 1 V voltage, with the resulting output being the current passing through the strain sensors. We selected a sampling frequency of 100 Hz and segmented the data into 10-s samples for detailed analysis. Our data collection process was specifically crafted to reflect real-world conditions, accommodating variations in the positioning and tightness of the collar with each use. Throughout our extensive data collection from various participants, we intentionally avoided strict calibration of the collar’s position or tightness. Participants were advised to wear the smart garment comfortably and put the collar around their necks, ideally positioned at the mid to upper throat level. This method ensured that our dataset represented a wide range of real-life scenarios, capturing the inherent variability in the collar positioning and tightness across different users and experimental setups.

### Sleep Conditions Dataset Collection.

The full study protocol was approved by the University of Cambridge Institutional Review Board (IRB) under the Engineering Department’s Ethical Approval for the Research Project: “Wearable Sensor System for Breath Monitoring.” All participants, comprising seven healthy students (average age 25, 4 males and 3 females) recruited from the University of Cambridge, provided informed consent. Since our subjects were healthy, they did not exhibit significant symptoms of Bruxism, CSA, or OSA. Therefore, the three types of sleep conditions were simulated following training under the guidance of medical experts. For the collection of bruxism, we included simulated instances of grinding, clenching, and tapping; for CSA, we instructed participants in voluntary end-expiratory central apnea during breathing; for the more challenging simulation of OSA, we trained participants to utilize Muller maneuver to maintain a lower intrathoracic pressure (44) and followed the characteristic descriptions of reports from the AASM, introducing clinical SpO_2_ as an auxiliary simulation tool, marking a segment as a valid OSA pattern only when SpO_2_ continuously fell below 90% within the sample, or a continuous decrease of more than 4% from baseline (45). These simulations were carefully developed with clinicians to ensure the primary mechanics of airway obstruction and breath maneuver conform realistic situations. The data for the other three conditions (nasal breath, mouth breath, and snoring) were collected during actual sleep states. To maintain consistency with the daily environments, sleep positions are freely chosen by the subjects. Each participant underwent a 1.5-h sleep data collection session, resulting in 2,329 10-s epochs. After processing, approximately 80% of the data were retained, with 20% filtered out due to instances where the participant had not entered a sleep state or where sleep states could not be confidently labeled. No unusable signals due to garment displacement or distortion were observed, demonstrating the robustness of the smart garment during natural sleep. In total, we obtained 2,119 usable samples, including 728 samples of nasal breath, 701 samples of mouth breath, 262 samples of snoring, 180 samples of bruxism, 102 samples of CSA, and 146 samples of OSA. See more detailed data collection and annotation protocol in *SI Appendix*, Note S2. The participants had varying body shapes, adding diversity to the study (see details in *SI Appendix*, Table S1). To mitigate class imbalance, proportional random sampling was employed to allocate 90% of the data for training and 10% for validation, ensuring an even distribution across all classes.

### Model Performance Evaluation.

The transfer learning accuracy was evaluated using the pruned version of SleepNet. After pruning and retraining the model on the original dataset, a fine-tuning process was conducted on two new participants’ data, utilizing a few-shot learning approach. The accuracy reported reflects the performance of this pruned and fine-tuned model. To ensure the robustness of the model and prevent overfitting, all testing was performed on an isolated testing set that was strictly separated from the training and validation datasets. This separation was maintained throughout the evaluation of both the original and pruned versions of SleepNet, including the transfer learning scenarios. The isolated testing approach ensures that performance metrics accurately reflect the model’s generalization capabilities under realistic conditions.

### Patient Data Validation.

To validate the system’s performance on real CSA and OSA cases, data were collected from two apnea patients (one diagnosed with CSA and one with OSA) under the approval of the Beijing Tongren Hospital Ethics Committee (Approval Number: TREC2022-KY110). The model, which was initially trained using simulated apnea data, was fine-tuned with patient data employing a few-shot learning approach utilizing 15 epoch samples. This resulted in an accuracy of 90.3%—a respectable performance, albeit lower than the 95% accuracy achieved in simulated scenarios (as shown in Fig. 5*C*). These findings demonstrate that the simulated signals provide meaningful reference characteristics for real-world applications while underscoring the necessity of further validation with larger and more diverse datasets.

### Software Environment.

Signal preprocessing was performed on a MacBook Pro equipped with an M1 Max CPU. Network training was conducted using Python 3.8.13, Miniconda 3, and PyTorch 2.0.1 in a performance-optimized environment. Training acceleration was enabled by CUDA on NVIDIA 4090 GPU.

## Supplementary Material

Appendix 01 (PDF)

Movie S1. A video summary of the main findings and paper.

## Data Availability

The datasets supporting this study are available in GitHub and can be accessed from https://github.com/tcy21414/Smart-Garment-for-Sleep-Monitoring (46).
