# Supplementary material for: A deep learning–enabled smart garment for accurate and versatile monitoring of sleep conditions in daily life
Source: Proc Natl Acad Sci U S A. 2025 Feb 11;122(7):e2420498122. doi: 10.1073/pnas.2420498122 (PMC11848432; doi:10.1073/pnas.2420498122)
Supplement: Supplementary file 1 — Appendix 01 (PDF) [file pnas.2420498122.sapp.pdf]

PNAS

Supporting Information for

A deep learning-enabled smart garment for accurate and versatile monitoring of sleep conditions in daily life.

Chenyu Tang[†1], Wentian Yi[†1], Muzi Xu[1], Yuxuan Jin[2], Zibo Zhang[1], Xuhang Chen[3], Caizhi Liao[1], Mengtian Kang[4], Shuo Gao[5], Peter Smielewski[3], Luigi G. Occhipinti[*1]

[1]Electrical Engineering Division, Department of Engineering, University of Cambridge, Cambridge CB3 0FA, United Kingdom
[2]The Cavendish Laboratory, Department of Physics, University of Cambridge, Cambridge CB3 0FZ, United Kingdom
[3]Department of Clinical Neurosciences, University of Cambridge, Cambridge CB2 0QQ, United Kingdom
[4] Department of Ophthalmology, Beijing Tongren Hospital, Capital Medical University, 100005 Beijing, China
[5]School of Instrumentation and Optoelectronic Engineering, Beihang University, 100191 Beijing, China

[†]These authors contributed equally: Chenyu Tang, Wentian Yi
*Correspondence to: Luigi G. Occhipinti.
Email: lgo23@cam.ac.uk

This PDF file includes:

Supporting text
Figures S1 to S27
Tables S1 to S3
Legend for Movie S1
SI References

Other supporting materials for this manuscript include the following:

Movie S1

Supporting Information Text

Supplementary Note 1 | Understanding Sleep Patterns and Their Physiological Manifestations

Sleep, occupying approximately one-third of an individual's daily life, profoundly influences overall human well-being. Monitoring sleep patterns has become crucial not only for enhancing sleep quality but also for early detection of chronic conditions like cardiovascular diseases. Among the various sleep patterns, nasal breathing, mouth breathing, snoring, bruxism, central sleep apnea (CSA), and obstructive sleep apnea (OSA) stand out as significant entities with distinct physiological manifestations and potential health implications [1-6].

1. Introduction to Sleep Patterns:

(1) Nasal Breathing: Nasal breathing, the natural and preferred mode of respiration during sleep, involves inhaling and exhaling through the nostrils. It facilitates air filtration, humidification, and temperature regulation, promoting efficient gas exchange in the lungs.

(2) Mouth Breathing: In contrast to nasal breathing, mouth breathing occurs when airflow bypasses the nasal passages, leading to inhalation and exhalation through the mouth. It may result from nasal congestion, structural abnormalities, or habitual behaviors, potentially affecting respiratory efficiency and oral health.

(3) Snoring: Snoring is characterized by turbulent airflow causing vibrations of the soft tissues in the upper airway during sleep. It often arises due to partial obstruction of the airway, leading to disrupted sleep architecture and daytime fatigue.

(4) Bruxism: Bruxism refers to the involuntary grinding, clenching, or tapping of teeth during sleep, often associated with stress, anxiety, or sleep disorders. It can lead to dental problems, temporomandibular joint dysfunction, and disrupted sleep.

(5) Central Sleep Apnea (CSA): CSA involves periodic cessation of airflow during sleep due to temporary failure of the brain to send signals to the respiratory muscles, yet without blockage in the airway. It disrupts normal breathing patterns and may be associated with neurological conditions or cardiac dysfunction.

(6) Obstructive Sleep Apnea (OSA): OSA is characterized by recurrent episodes of complete or partial upper airway obstruction during sleep, leading to breathing pauses and oxygen desaturation. It is commonly associated with obesity, anatomical factors, and increased risk of cardiovascular morbidity.

2. Significance of Monitoring Sleep Patterns:

- Each sleep pattern holds implications for overall health, with potential adverse effects on various physiological systems.

- Nasal and mouth breathing patterns can impact respiratory function, oral health, and sleep quality.

- Snoring, bruxism, CSA, and OSA are linked to cardiovascular diseases, metabolic disorders, and emotional disturbances, highlighting the importance of early detection and intervention.

3. Physiological Manifestations and Pathophysiology:

- These sleep patterns manifest through vibrations and disturbances in physiological structures such as the velum, oropharynx, tongue, and epiglottis.

- Vibrations generated by airflow turbulence, muscle contractions, or neurological abnormalities propagate through the extrinsic laryngeal muscles, leading to audible sounds and physiological responses.

- Understanding the mechanisms underlying these vibrations and their propagation pathways is essential for accurate detection and monitoring of sleep patterns.

Supplementary Note 2 | Protocol for Data Collection and Annotation

In this section, we provide a detailed protocol for the collection and annotation of sleep behavior data, ensuring adherence to clinical standards and facilitating acceptance by reviewers with medical backgrounds. The protocol encompasses the simulation of various sleep behaviors, including bruxism, central sleep apnea (CSA), and obstructive sleep apnea (OSA), along with the collection of data for nasal breathing, mouth breathing, and snoring.

1. Data Collection Setup:

Participants: Healthy students recruited from the University of Cambridge (7 students, average age 25, 4 males and 3 females).

Equipment: Video recording device for capturing behavioral cues, audio recording device for snoring detection, and auxiliary sensor for physiological measurements (clinical SpO_2 reader). All data collection was performed in a supine position.

2. Collection of Nasal and Mouth Breathing Data:

Participants completed questionnaires assessing habitual breathing patterns and underwent video recording during sleep. Video footage was reviewed to confirm breathing mode (nasal vs. mouth) during designated epochs.

3. Collection of Snoring Data:

Simultaneous audio recording during sleep allowed for the identification of snoring events. Snoring episodes were annotated based on sound frequency, duration, and intensity.

4. Simulation and Collection of Bruxism Data:

Participants were instructed to simulate bruxism behaviors, including grinding, clenching, and tapping, while being monitored. Each behavior was demonstrated by a medical expert and practiced by participants before data collection.

5. Simulation and Collection of CSA Data:

Participants were instructed in voluntary end-expiratory central apnea during specific breathing cycles while being monitored. Each simulation had a wash-out phase of at least 60s to ensure a complete recovery in metabolism. These simulated apneic episodes characteristic of central sleep apnea, aiming to replicate physiological responses observed during clinical assessments.

6. Simulation and Collection of OSA Data:

- Participants were instructed to execute Muller maneuver to maintain a lower intrathoracic pressure during sleep. The simulation process adhered to the characteristic descriptions of obstructive sleep apnea (OSA), including the patterns of SpO_2 levels, as outlined by the American Academy of Sleep Medicine (AASM) [7]. Each simulation had a wash-out phase of at least 60s to ensure a complete recovery in metabolism [8].

- SpO_2 levels are continuously monitored to verify the success of the OSA simulation. An episode is considered a valid simulation of OSA if it meets one of the following criteria: a continuous decrease in SpO_2 of more than 4% from baseline, or a reduction in SpO_2 to below 90% within any given epoch. This dual criterion approach provides a comprehensive capture of the physiological features of OSA and enables more accurate data for subsequent analysis.

7. Data Annotation and Quality Assurance:

- Annotators, including trained researchers and medical professionals, reviewed collected data to ensure accuracy and consistency in labeling sleep behaviors.

- Annotation criteria aligned with established clinical guidelines and were verified through consensus among annotators.

8. Summary of Data Collection:

A total of 2119 epochs were collected, comprising 728 epochs of nasal breathing, 701 epochs of mouth breathing, 262 epochs of snoring, 180 epochs of bruxism, 102 epochs of CSA, and 146 epochs of OSA.

By adhering to this protocol, we aimed to simulate and collect representative data sets encompassing diverse sleep behaviors, facilitating the development and validation of our sleep monitoring system within the constraints of ethical and practical considerations. Supplementary Figure 20 shows the environmental setup of the data collection site.

Supplementary Note 3 | Offline Analysis vs Real-Time Application

This section highlights the considerations and adaptations made to transition our sleep monitoring system from offline analysis to potential real-time applications. The following points are detailed, supported by the Fig. S27:

1. Epoch Labeling Strategy:

In offline analysis, sleep condition epochs were labeled based on the most health-critical state present, ensuring prioritization of high-risk conditions. For instance, epochs with overlapping conditions, such as nasal breathing transitioning into central sleep apnea (CSA), were labeled as CSA due to its severe health implications. Supplementary Figure a visualizes this labeling approach.

2. Real-Time Sliding Window Mechanism:

For real-time application, a sliding window approach using 10-second intervals is proposed. This mechanism allows for continuous monitoring and detection of sleep states within overlapping windows. By ensuring a dynamic yet robust framework, this strategy minimizes the risk of missing transient but harmful states.

3. Health-Centric Prioritization:

Sleep conditions are categorized by their health impact severity:

High-Risk Conditions: CSA and OSA, associated with hypoxemia, cardiovascular strain, and neurocognitive impairments.

Moderate Impact: Mouth breathing and snoring, indicative of airway compromise.

Low Impact: Bruxism, mainly affecting dental health and temporomandibular disorders.

This hierarchy underscores the clinical relevance of detecting harmful states promptly, even in the presence of transient benign conditions (e.g., Fig. S27 b).

4. Limitations of Transition Detection:

Boundary transitions, such as shifts between nasal and mouth breathing or bruxism to apnea, may lead to short-term classification discrepancies. However, the proposed real-time design ensures these transitions are accounted for in subsequent overlapping windows, improving overall robustness.

The above mechanisms ensure a seamless adaptation of the offline-tested model to real-time scenarios, aligning with practical healthcare requirements. Supplementary Figure illustrates examples of epoch labeling and real-time transitions in simulated scenarios.

Supplementary Note 4 | Prototype Setup and Future Wireless Integration

During the testing phase, the smart garment prototype was connected via external cables for data acquisition purposes. This setup was chosen for the convenience of prototyping and to facilitate detailed characterization of the system's performance. However, it is important to note that this wired setup does not represent the intended final form of the smart garment. The system is fully compatible with wireless integration, which would eliminate the need for external cables in real-world applications.

The device characterization in Fig. 2 demonstrate the robust performance of the printed strain sensors on the textile substrate, showcasing their stability and sensitivity under strain. These good printing charateristics are a critical enabler for integrating advanced printed electronics technologies, such as lightweight and flexible wireless modules, directly onto garments. Recent advancements in printed electronics have demonstrated the feasibility of embedding wireless readout modules on textile substrates, leveraging techniques such as screen printing, inkjet printing, or roll-to-roll manufacturing. These modules are capable of wirelessly transmitting high-resolution data to external devices such as smartphones or cloud platforms for further analysis. This approach is supported by well-established technologies in the field [11-13].

Furthermore, the garment itself has been specifically designed with comfort in mind. The use of elastic, breathable fabrics ensures that the smart garment provides a high level of user comfort, making it suitable for long-term, daily wear. This aspect is particularly important as the system aims to serve as a versatile sleep monitoring platform for a wide range of users, including both healthy individuals and patients. By focusing on the garment's intrinsic comfort and ensuring compatibility with wireless readout modules, we address both usability and scalability for future commercialization or large-scale clinical trials.

The primary goal of this work is to demonstrate the feasibility and effectiveness of strain sensing as a single-modality solution for versatile sleep monitoring. The integration of wireless modules is a natural next step in the system's development and will further enhance user comfort while maintaining the system's accuracy and robustness. Future iterations of the garment will incorporate these wireless advancements, enabling a seamless user experience without the constraints of external cables.

Fig. S1. a, Stretching simulation setup for a PUA-isolated textile strain sensing array, with over 40% uniaxial strain applied to the textile. **b-f,** Results of the stretching simulation with PUA at different Young's modulus levels: (b) 2 GPa, (c) 1.5 GPa, (d) 1 GPa, (e) 0.5 GPa, (f) 0.25 GPa

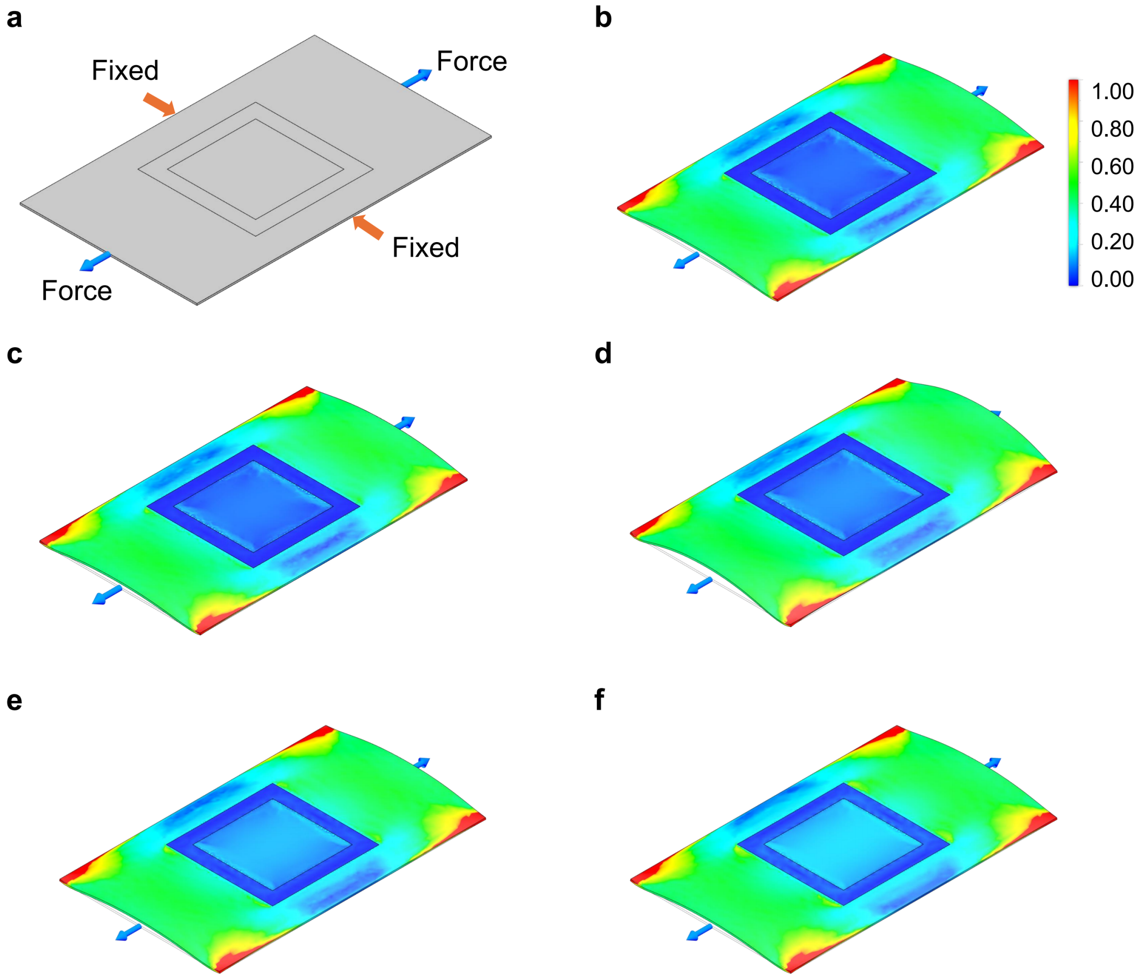

Fig. S2. Simulated strain at the center of the textile strain sensor array isolated with PUA at different Young's modulus levels.

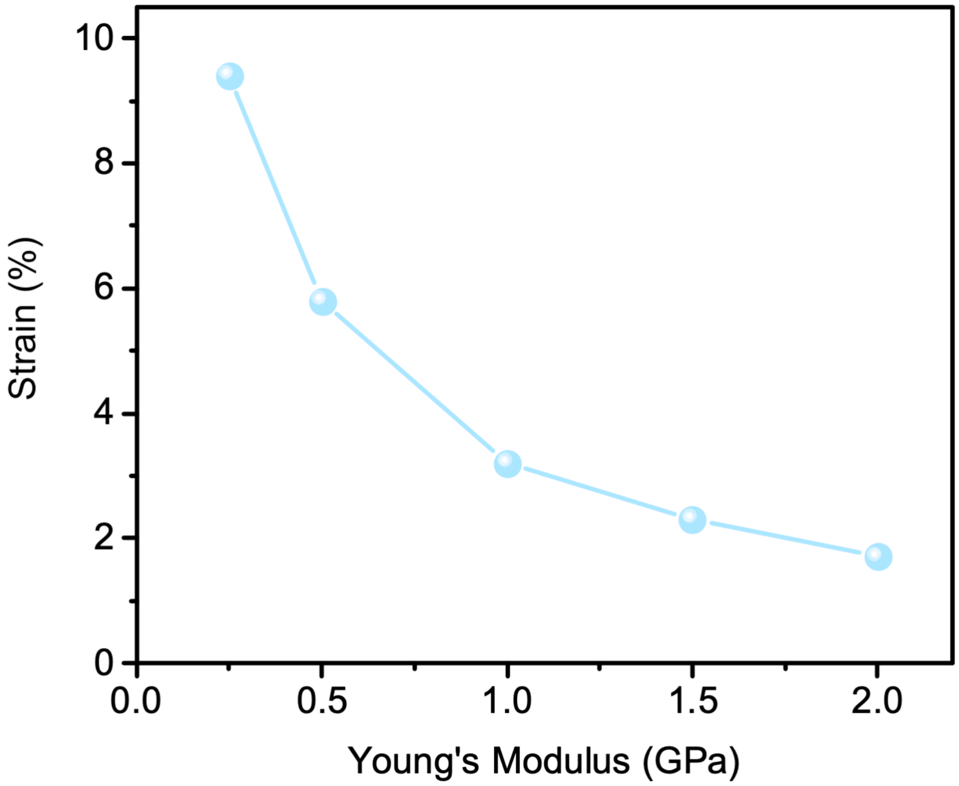

Fig. S3. a, Sensing simulation setup for the PUA-isolated textile strain sensing array, with the Young's modulus of PUA set at 1 GPa. A 1 N force is applied by a ball to the center of the array to simulate skin contact. **b,** Result of the simulation reveals 40% uniaxial strain isolated from the array, with significant localized strain of 4.8% at the center where the ball contacts, while other sensing areas show a 2.6% strain.

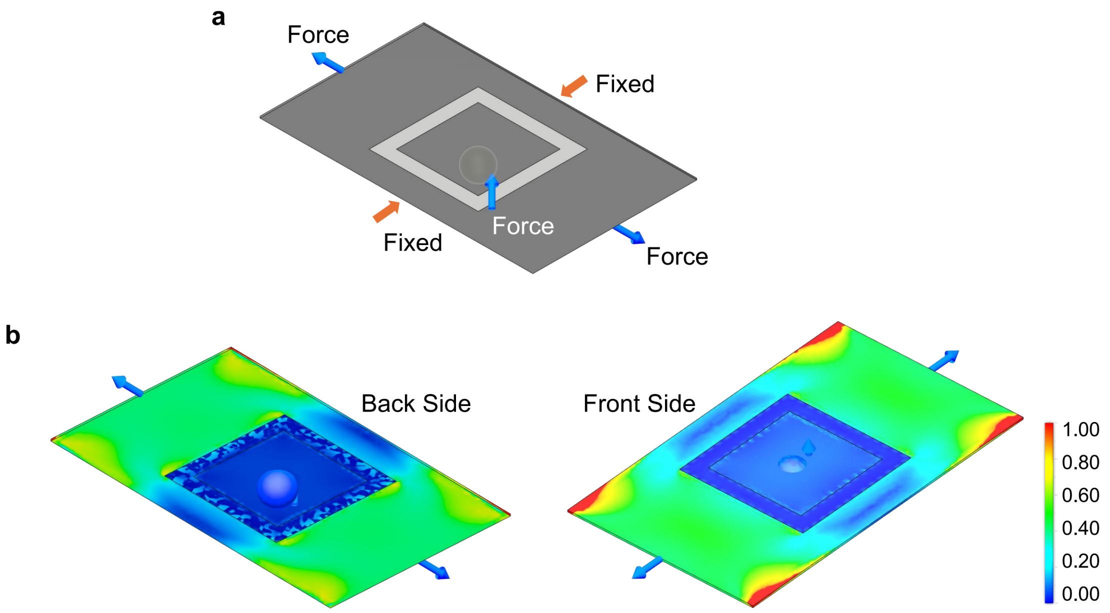

Fig. S4. AFM of **a,** graphene/EC composite and **b,** only graphene on Si/SiO2 substrate. EC was removed by washing with 2:8 v EtOH/toluene.

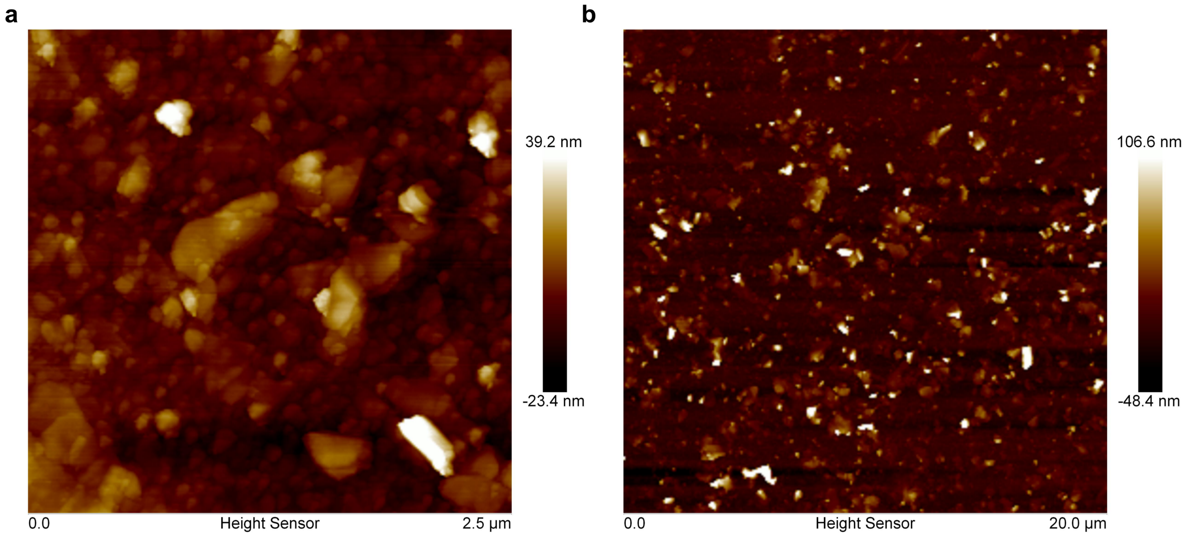

Fig. S5. Illustration of the ink penetration with (top) and without (bottom) CMC Na starching layer.

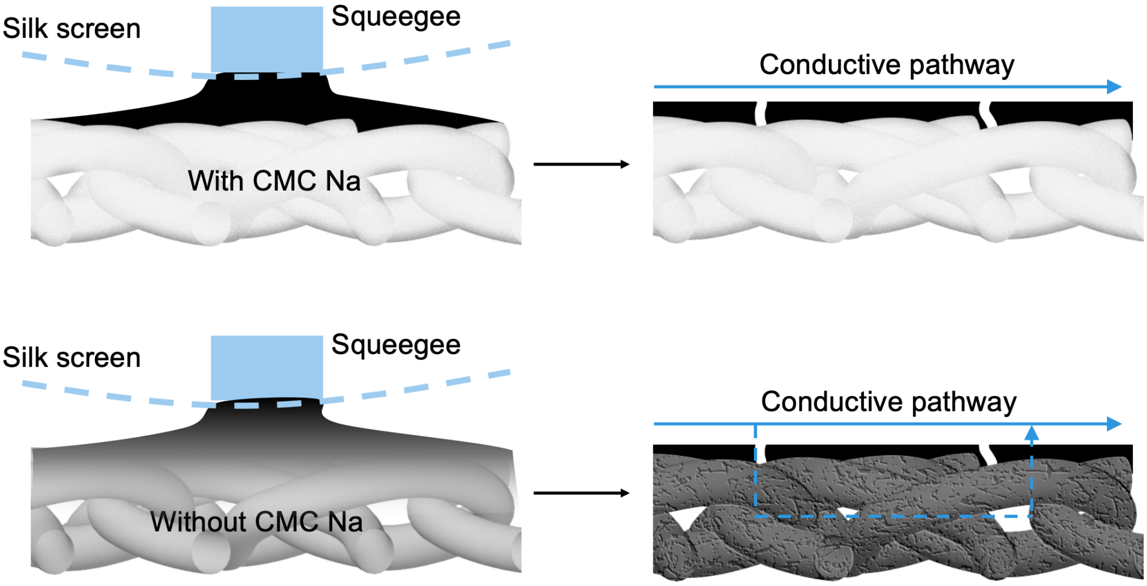

Fig. S6. a, SEM image of stretched textile with graphene/EC coating at 5% strain. **b,** SEM image of stretched textile with graphene/EC coating at 10% strain.

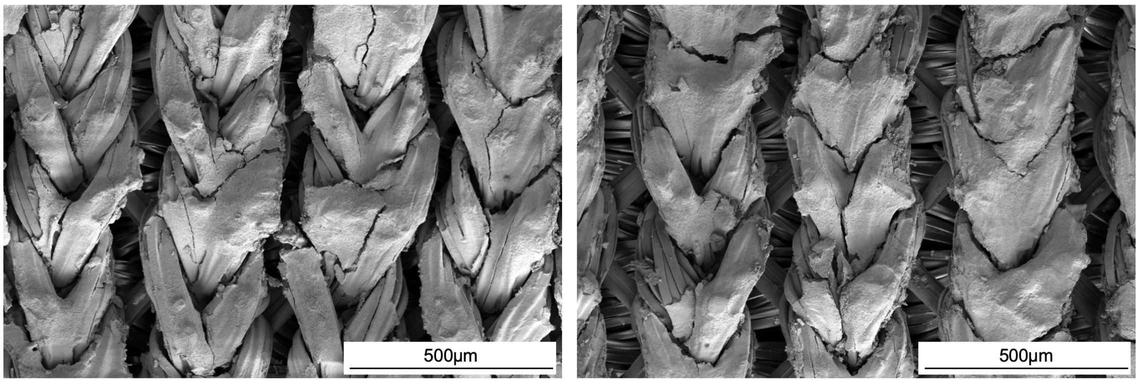

Fig. S7. a, SEM of textile directly printed with the graphene ink. **b,** SEM of textile treated with CMC Na solution and printed with graphene ink. Dashed boxes label the ink penetration depth in each sample.

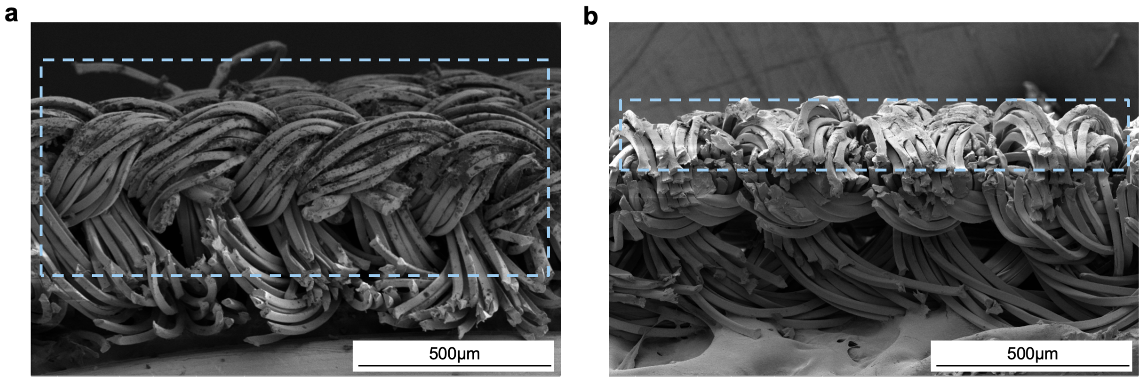

Fig. S8. Schematic of the printing process. The number of prints refers to the number of cycles the substrate is printed for. During each cycle, the silk frame is lowered to the substrate, the flood blade travels backward to spread the ink, the squeegee travels to print, and the silk frame is raised to leave the substrate.

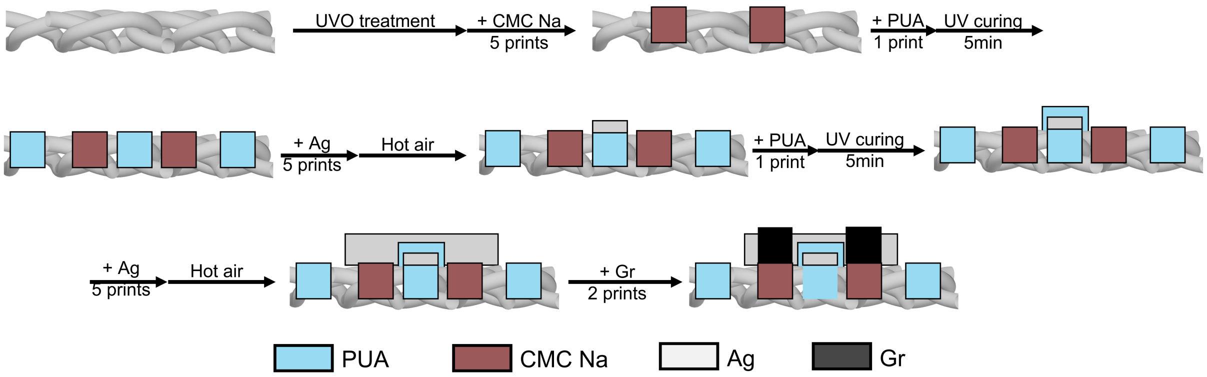

Fig. S9. Breathability test. Comparison of pure textile and the textile substrate printed with the strain sensor array in the moisture vapor transmission rate (MVTR) test (20 °C, 59% RH). There is no decrease of the breathability after printing the sensor array on the textile substrate. The printed textile strain sensor array shows higher breathability (~20,000 $g/m^2/24h$) than Tegaderm (800~2000 $g/m^2/24h$), a commonly used commercially available medical dressing by 3M [9].

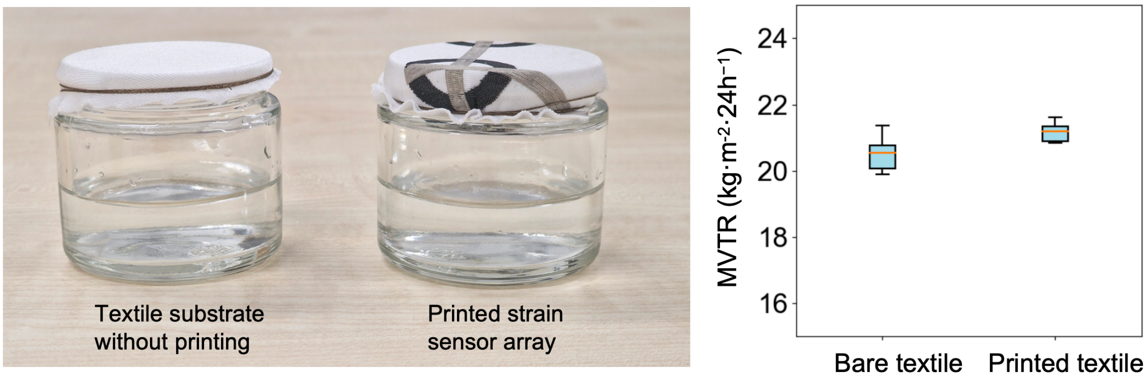

Fig. S10. Biocompatibility test. Comparison of wearing the textile sensor and Ecoflex substrate for 8 hours. No sign of irritation or side effect in the textile sensor area. Skin irritation appeared in the Ecoflex area.

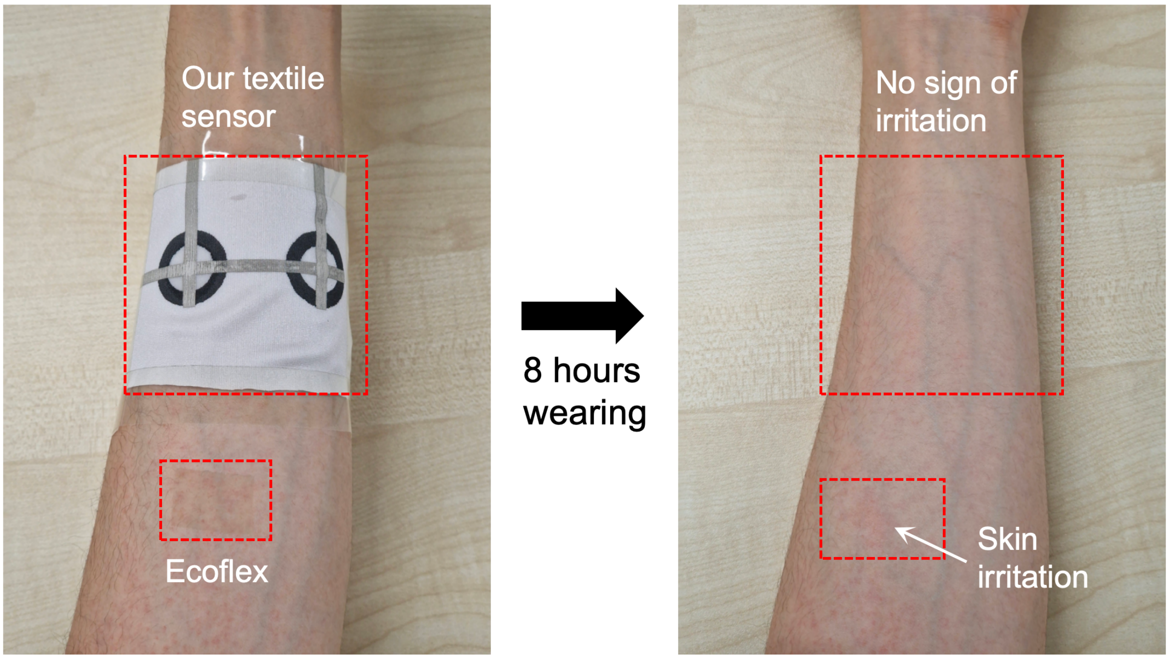

Fig. S11. Sweat environment test on smart garment sensing area. The artificial sweat solution was prepared according to ISO 3160-2 standard, consisting of 20 g/L sodium chloride (NaCl), 17.5 g/L ammonium chloride (NH4Cl), 5 g/L urea, 2.5 g/L acetic acid (CH3COOH), 15 g/L lactic acid, and 80 g/L sodium hydroxide (NaOH) for pH adjustment, and deionized water. The pH of the solution was adjusted to 4.7 using NaOH. Sweat volumes were calculated based on the surface area of the smart garment's sensing area, with minimum (0.32 mg/cm²/min) and maximum (2.7 mg/cm²/min) sweat rates applied over a 10-minute period [10]. The calculated artificial sweat was evenly distributed across the sensing area using a pipette. The garment was in the environment of 20 °C, 59% RH for the duration of the test.

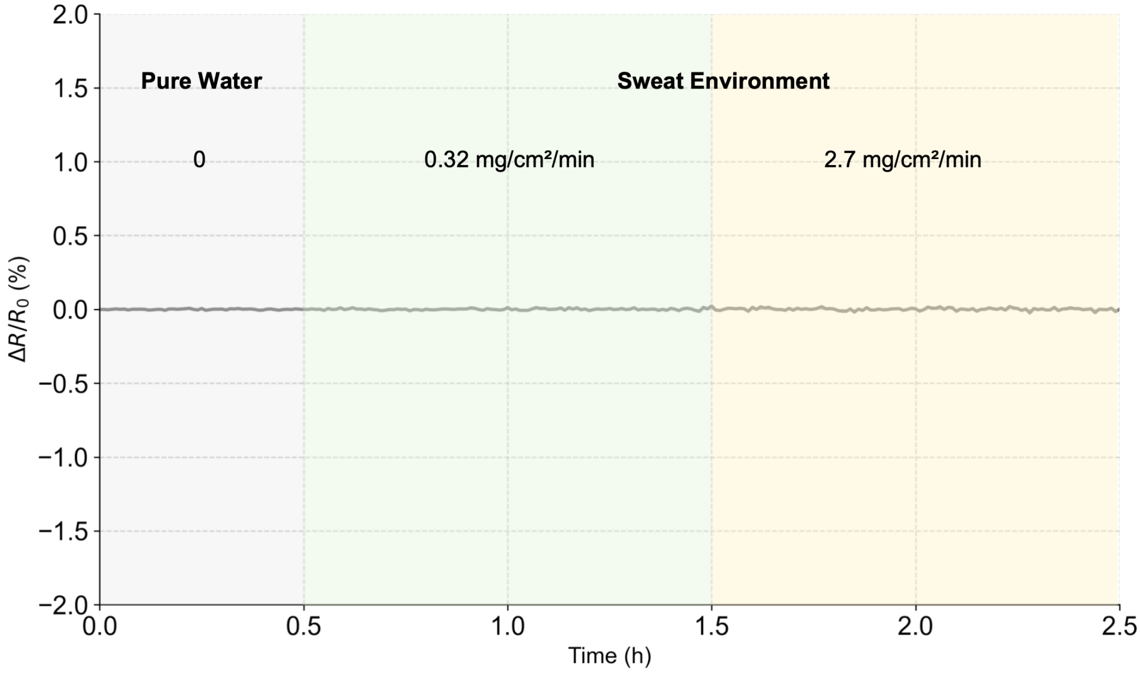

Fig. S12. Analysis of the signal power density and correlation between different channels when the strain sensor array is worn at different positions. The red triangle represents the center of the throat, which serves as a reference position.

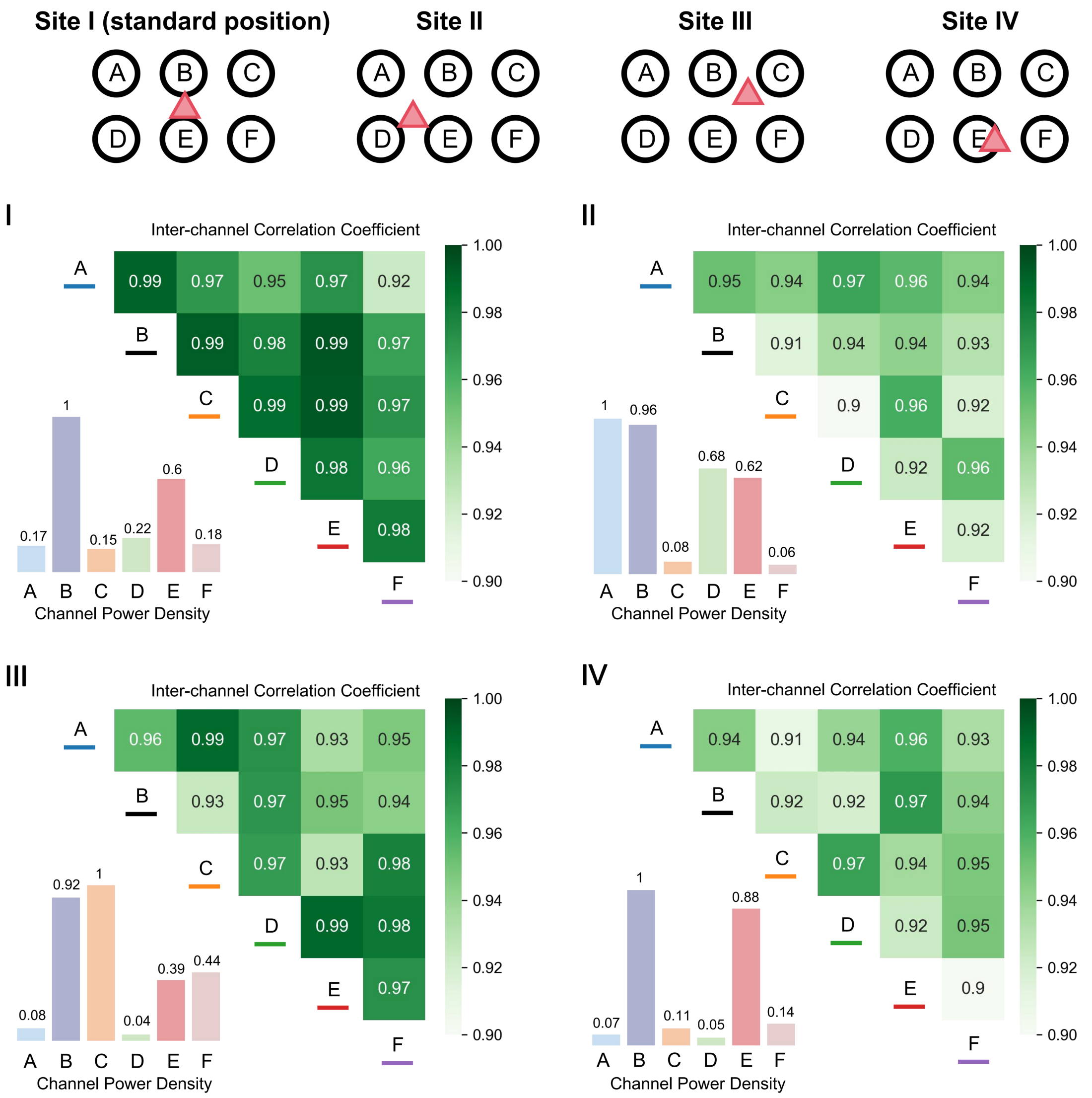

Fig. S13. Typical samples of nasal breathing. The Time-frequency spectrogram represents the channel with the strongest response.

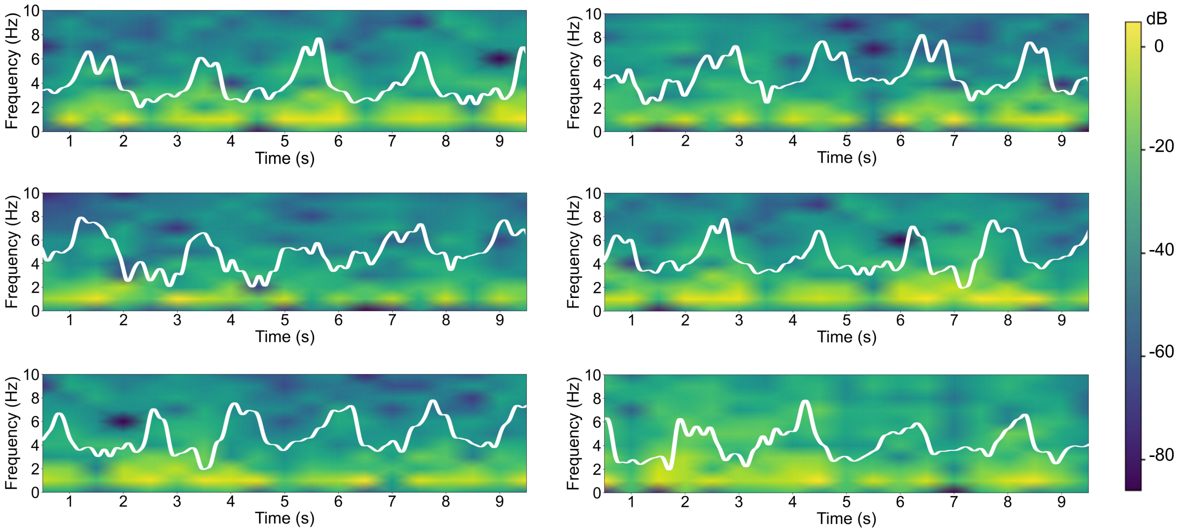

Fig. S14. Typical samples of mouth breathing. The Time-frequency spectrogram represents the channel with the strongest response.

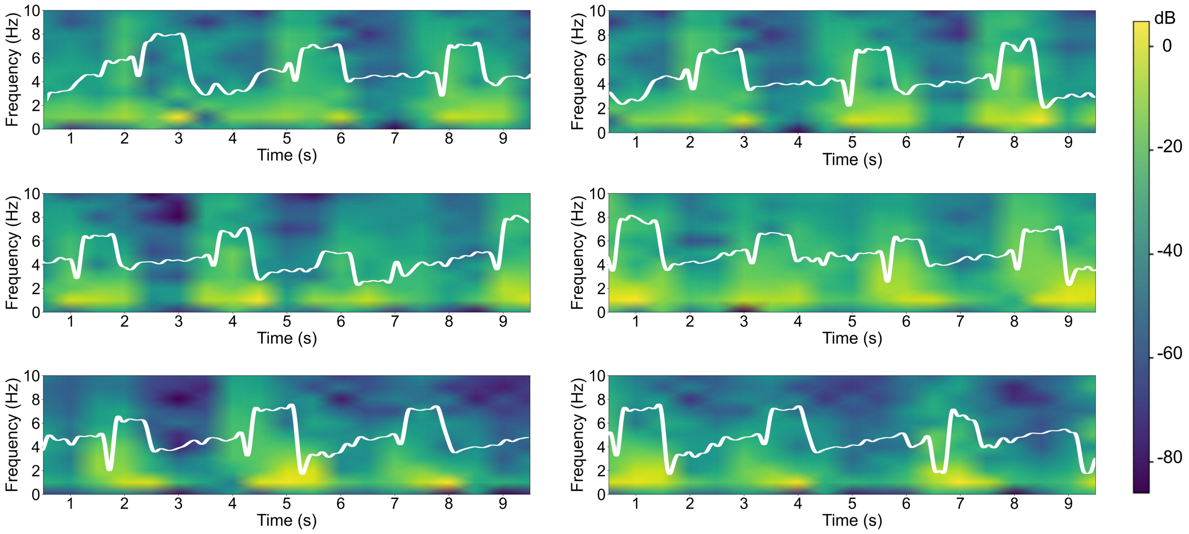

Fig. S15. Typical samples of snoring. The Time-frequency spectrogram represents the channel with the strongest response.

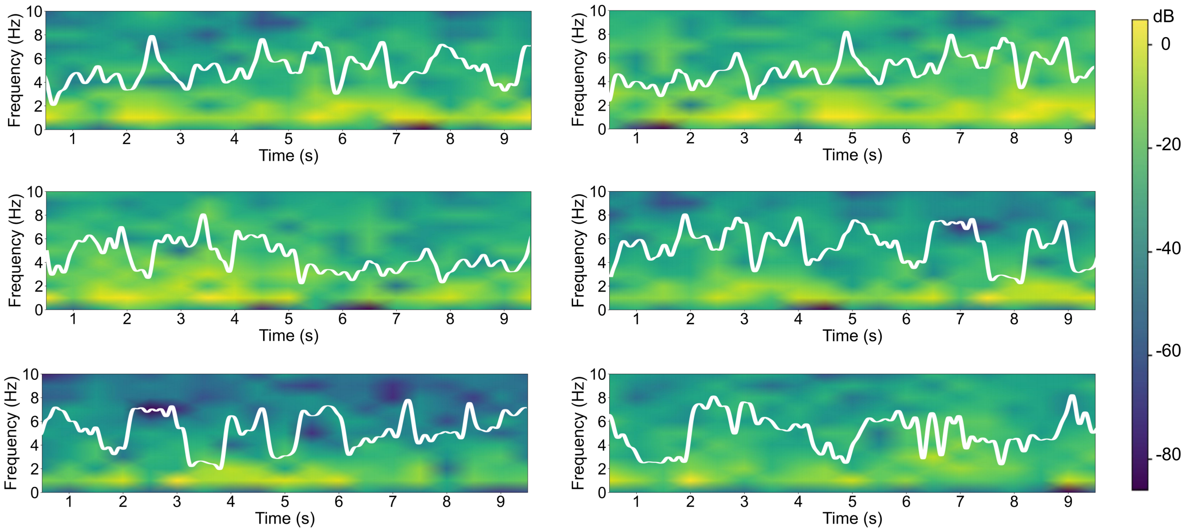

Fig. S16. Typical samples of bruxism. The Time-frequency spectrogram represents the channel with the strongest response.

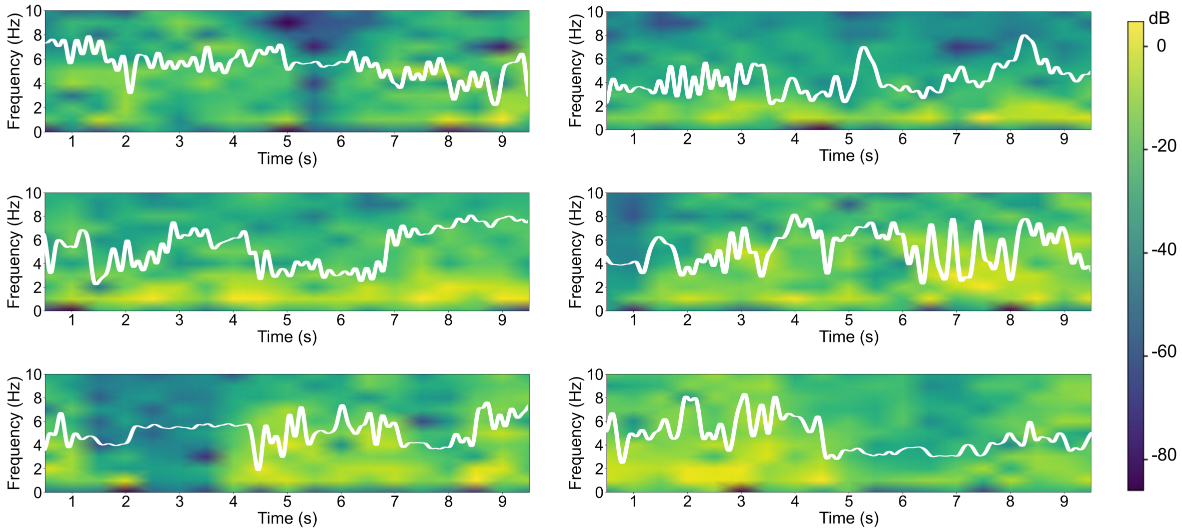

Fig. S17. Typical samples of CSA. The Time-frequency spectrogram represents the channel with the strongest response.

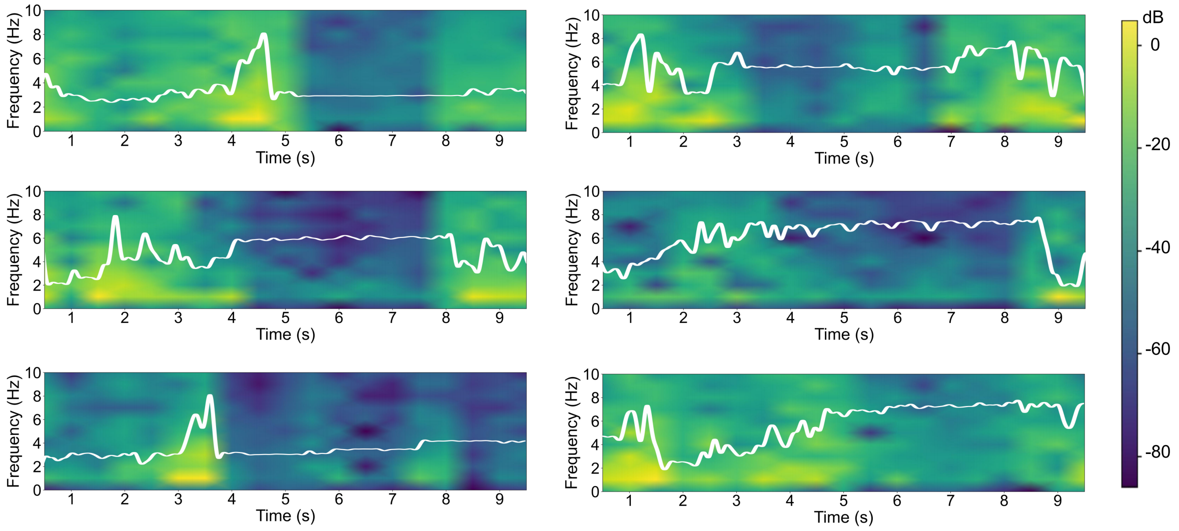

Fig. S18. Typical samples of OSA. The Time-frequency spectrogram represents the channel with the strongest response.

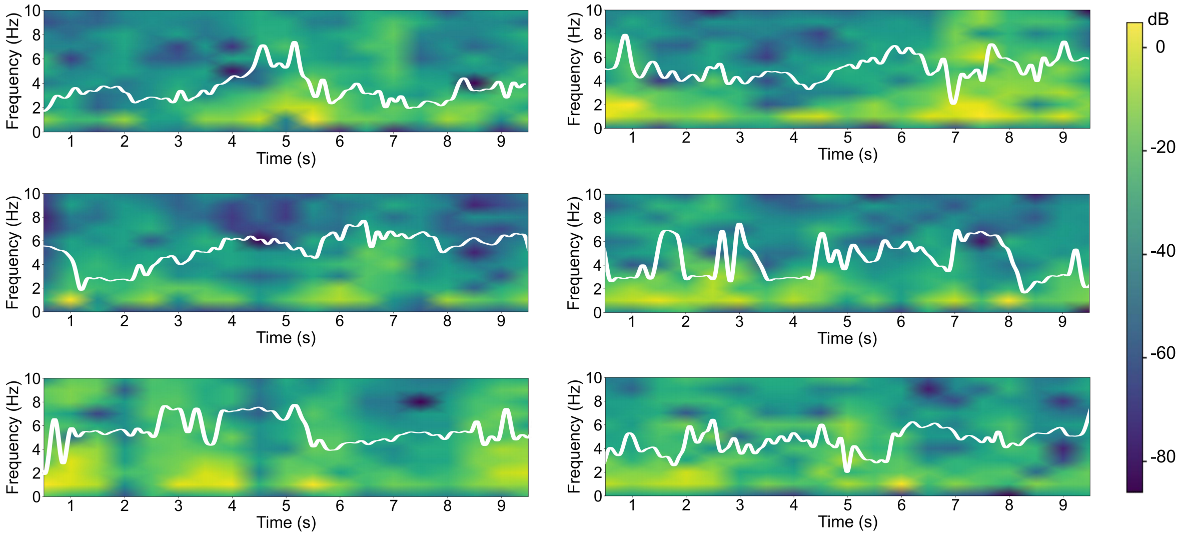

Fig. S19. Performance of the baseline models. The comparative experiments of six baseline models were conducted on the dataset. **a-f** correspond to the train-validation curves and confusion matrices of the six models under their optimal hyperparameters after random optimization.

a

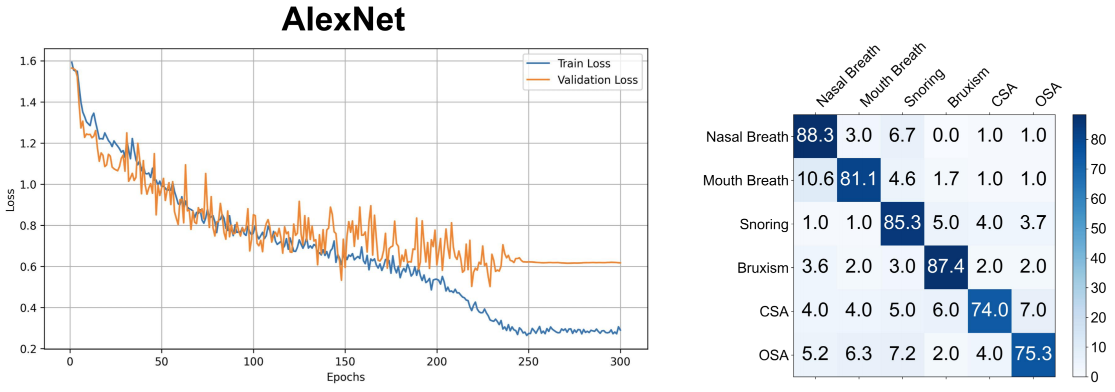

b

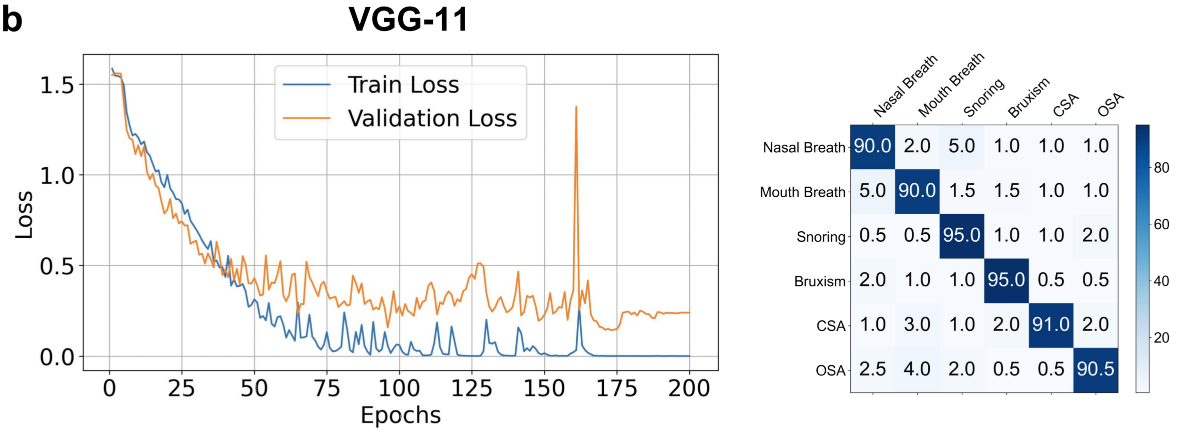

c

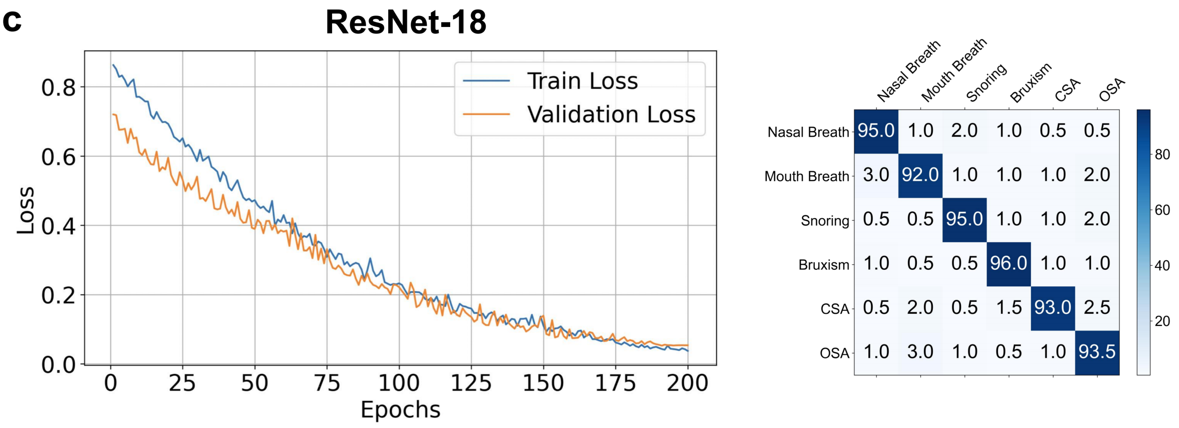

d

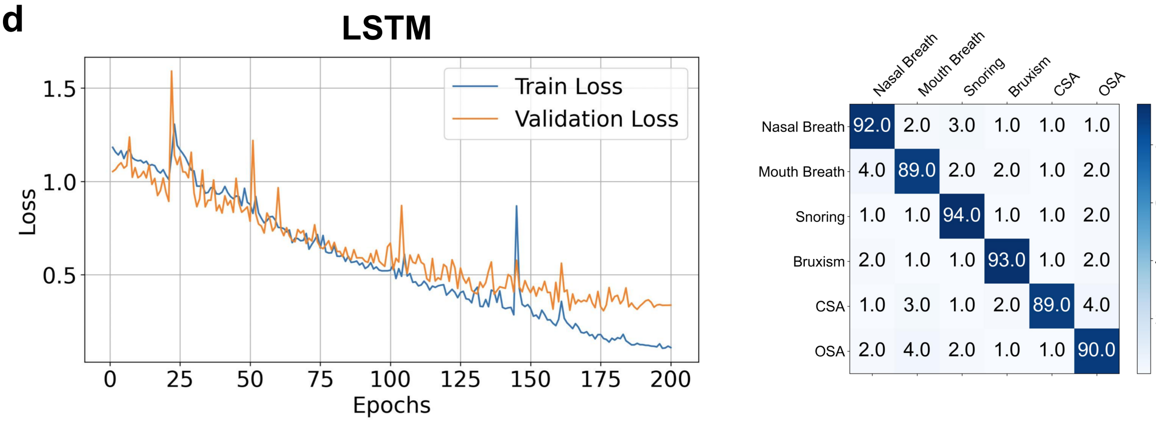
LSTM
Train Loss
Validation Loss
Loss
Epochs
Nasal Breath
Mouth Breath
Snoring
Bruxism
CSA
OSA
Nasal Breath 92.0 2.0 3.0 1.0 1.0 1.0
Mouth Breath 4.0 89.0 2.0 2.0 1.0 2.0
Snoring 1.0 1.0 94.0 1.0 1.0 2.0
Bruxism 2.0 1.0 1.0 93.0 1.0 2.0
CSA 1.0 3.0 1.0 2.0 89.0 4.0
OSA 2.0 4.0 2.0 1.0 1.0 90.0

e

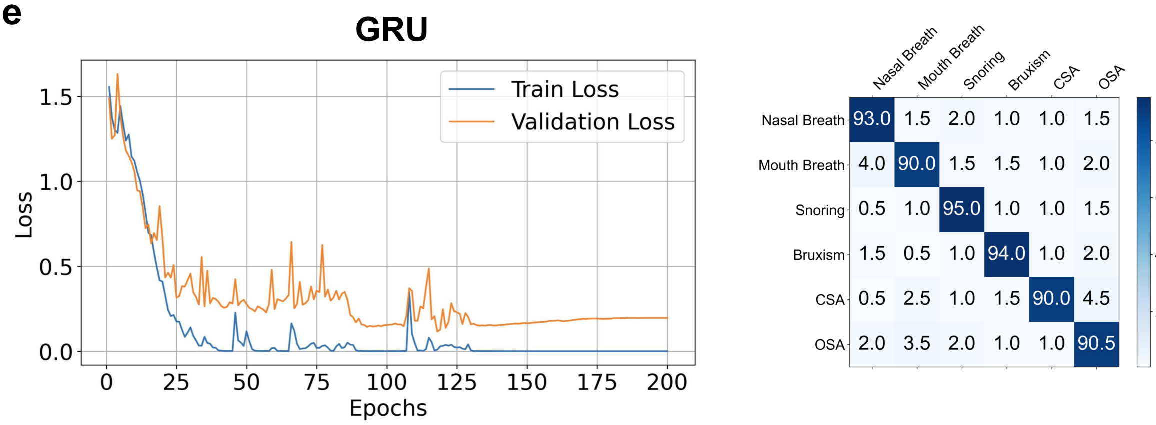
GRU
Train Loss
Validation Loss
Loss
Epochs
Nasal Breath
Mouth Breath
Snoring
Bruxism
CSA
OSA
Nasal Breath 93.0 1.5 2.0 1.0 1.0 1.5
Mouth Breath 4.0 90.0 1.5 1.5 1.0 2.0
Snoring 0.5 1.0 95.0 1.0 1.0 1.5
Bruxism 1.5 0.5 1.0 94.0 1.0 2.0
CSA 0.5 2.5 1.0 1.5 90.0 4.5
OSA 2.0 3.5 2.0 1.0 1.0 90.5

f

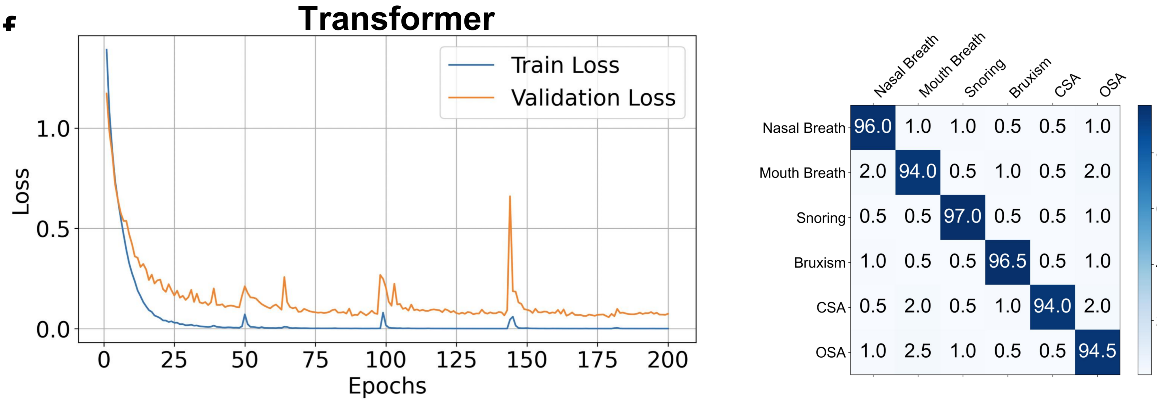
Transformer
Train Loss
Validation Loss
Loss
Epochs
Nasal Breath
Mouth Breath
Snoring
Bruxism
CSA
OSA
Nasal Breath 96.0 1.0 1.0 0.5 0.5 1.0
Mouth Breath 2.0 94.0 0.5 1.0 0.5 2.0
Snoring 0.5 0.5 97.0 0.5 0.5 1.0
Bruxism 1.0 0.5 0.5 96.5 0.5 1.0
CSA 0.5 2.0 0.5 1.0 94.0 2.0
OSA 1.0 2.5 1.0 0.5 0.5 94.5

Fig. S20. The environmental setup of the data collection site. The data collection environment is set up to simulate an actual at-home sleep environment. A camera continuously records video aimed at the participant's face. Together with questionnaires, it provides annotations for three sleep states: nasal breathing, mouth breathing, and snoring. SpO_2 is used as a reference to verify the validity of the simulation for OSA. During the actual test, additional wires are required to connect the smart garment and the readout module.

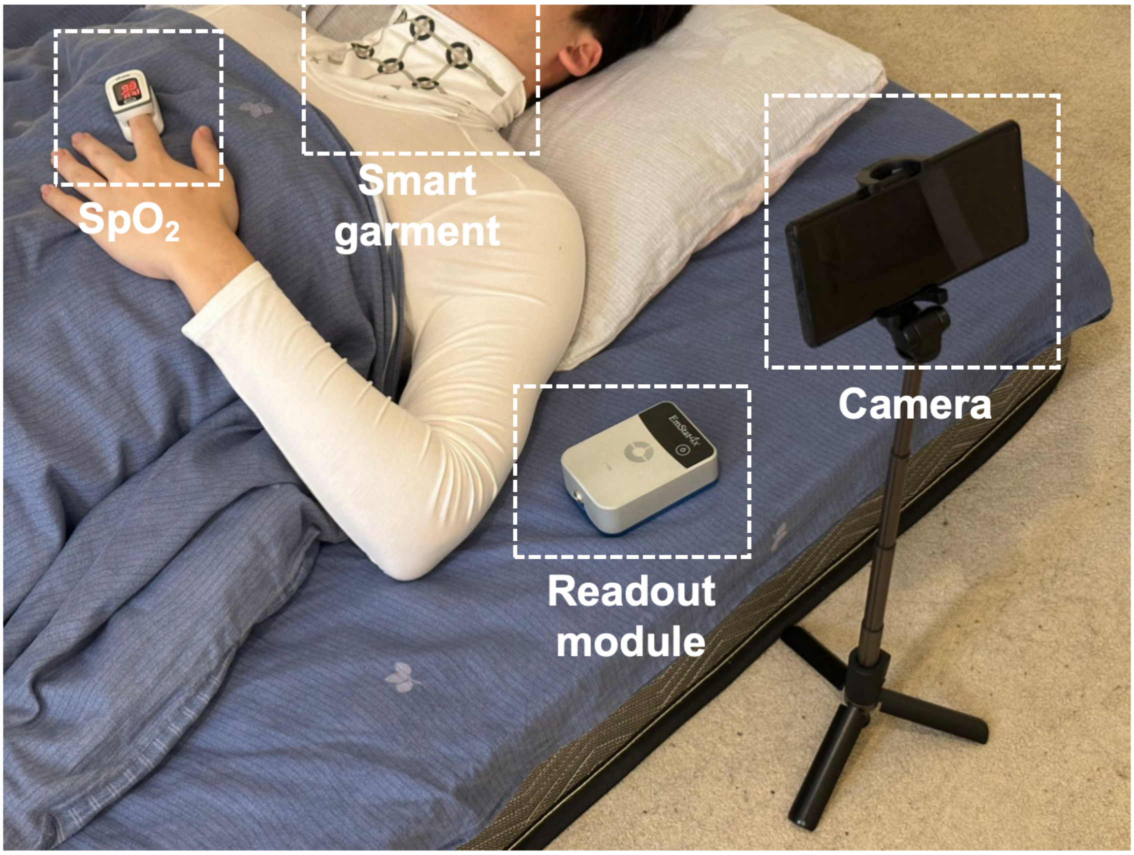

Fig. S21. Comparison of sensor configurations for classification accuracy across six sleep states. a, 2-Channel Configuration: Limited spatial coverage resulted in lower accuracy (84.1%). **b,** 4-Channel Configuration: Improved accuracy (91.7%), but misclassifications were still observed under complex conditions. **c,** 6-Channel Configuration: Achieved the highest classification accuracy (98.6%), demonstrating robust detection across all sleep states. Increasing the sensor count beyond six offers minimal accuracy improvement while increasing energy consumption and computational complexity.

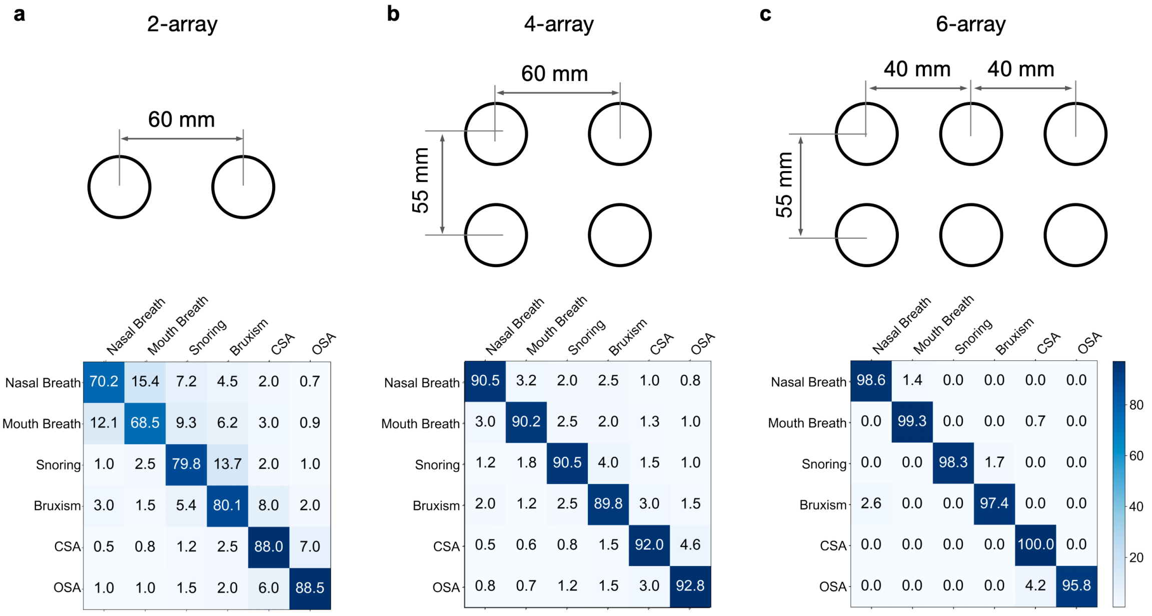

Fig. S22. User feedback on the comfort of the smart garment during overnight use. Participants rated the device on five dimensions of comfort after overnight wearing: Fit (how well the garment conforms to the neck), Breathability (airflow and heat dissipation), Flexibility (ease of movement), Skin Friendliness (absence of irritation), and Overall Comfort (general satisfaction), using a 5-point Likert scale (1 = very uncomfortable, 5 = very comfortable). The radar chart shows that all dimensions received average scores above 4.0, indicating that the device provides a high level of comfort for overnight sleep monitoring. These results demonstrate the smart garment's practicality and comfort, even during extended use.

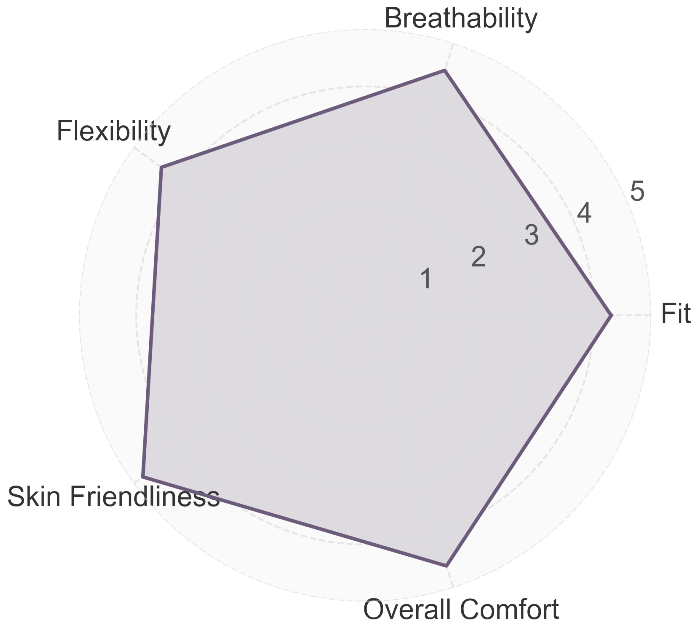

Fig. S23. Simulated strain distribution results for different neck circumferences. Strain distribution in the isolated sensing area (εis) and the substrate (εsub) was simulated for neck circumferences of 25 cm, 30 cm, and 35 cm under a 5 cm uniaxial tensile stretch. The isolated sensing area exhibits strain levels consistently below 1%, demonstrating the effectiveness of the strain isolation strategy in mitigating strain variability caused by different neck circumferences. These results highlight the robustness of the sensor design for wearable applications across a range of users.

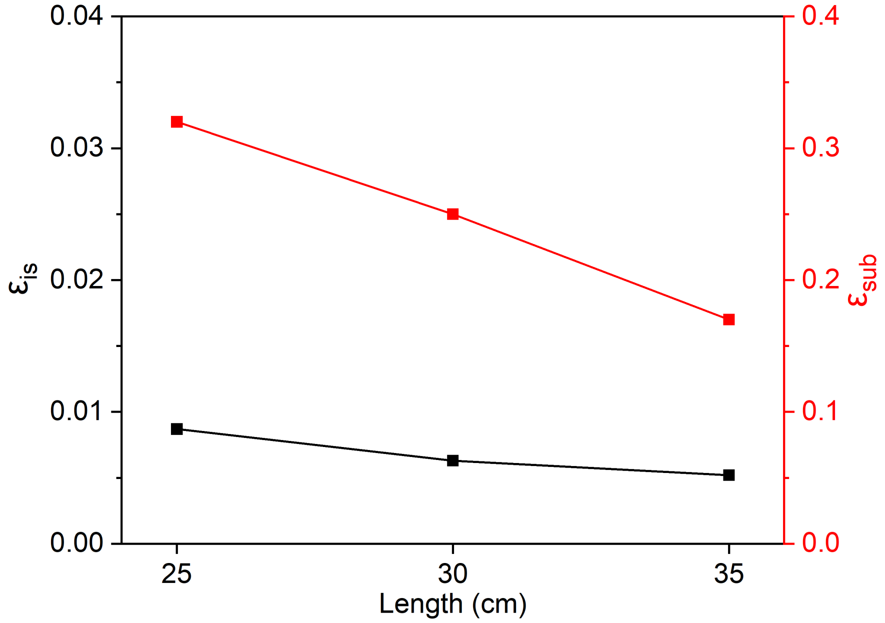

Fig. S24. Aging test of a textile-based graphene strain sensor over 28 days. The sensor was measured daily using a potentiostat to record the conductivity of the graphene coating between the x and y electrodes and compare it with the initial conductivity value.

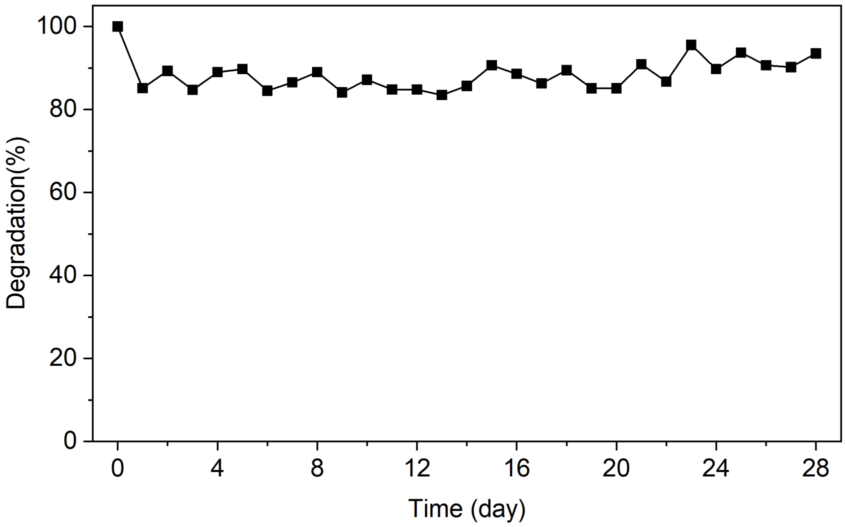

Fig. S25. Hysteresis of the relative resistance change during a loading/unloading cycle.

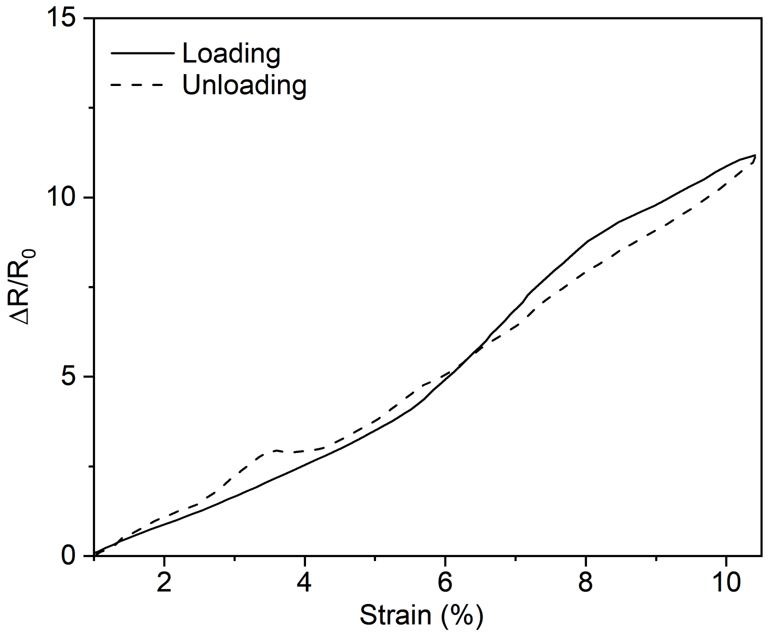

Fig. S26. Storage modulus spectrum of the textile-based graphene sensor at room temperature. The storage modulus remains stable and high across the 1–100 Hz frequency range, indicating that the sensor substrate is primarily elastic with minimal viscoelastic deformation. This ensures precise strain response without significant energy dissipation, extending the sensor's effective bandwidth beyond 100 Hz.

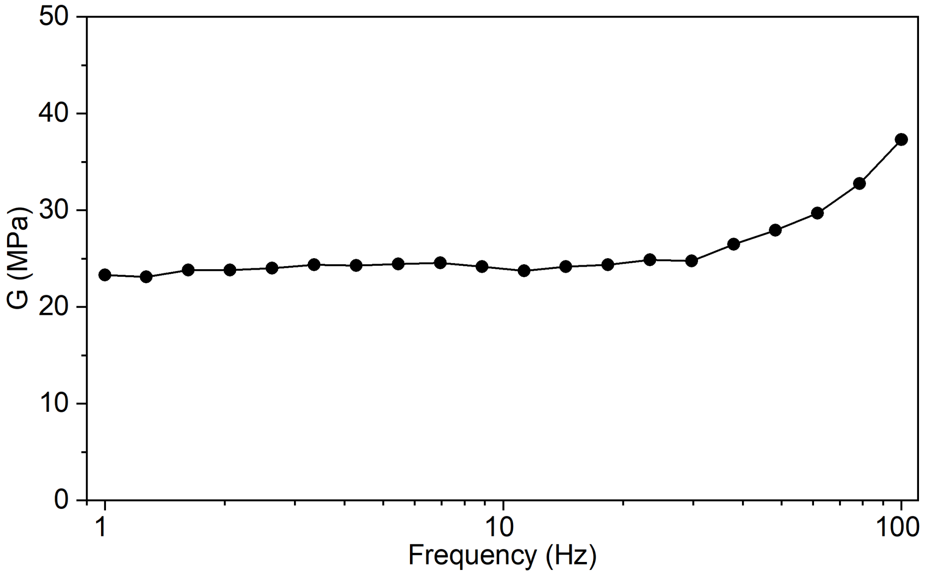

Fig. S27. Illustration of the labeling strategy and real-time application framework for sleep condition detection. a, Health-centric labeling strategy: Epochs are labeled based on the most health-critical condition present, ensuring prioritization of harmful states. **b,** Real-time application framework: A 10-second sliding window approach enables continuous monitoring, capturing high-risk conditions during transitions while balancing detection resolution and practicality.

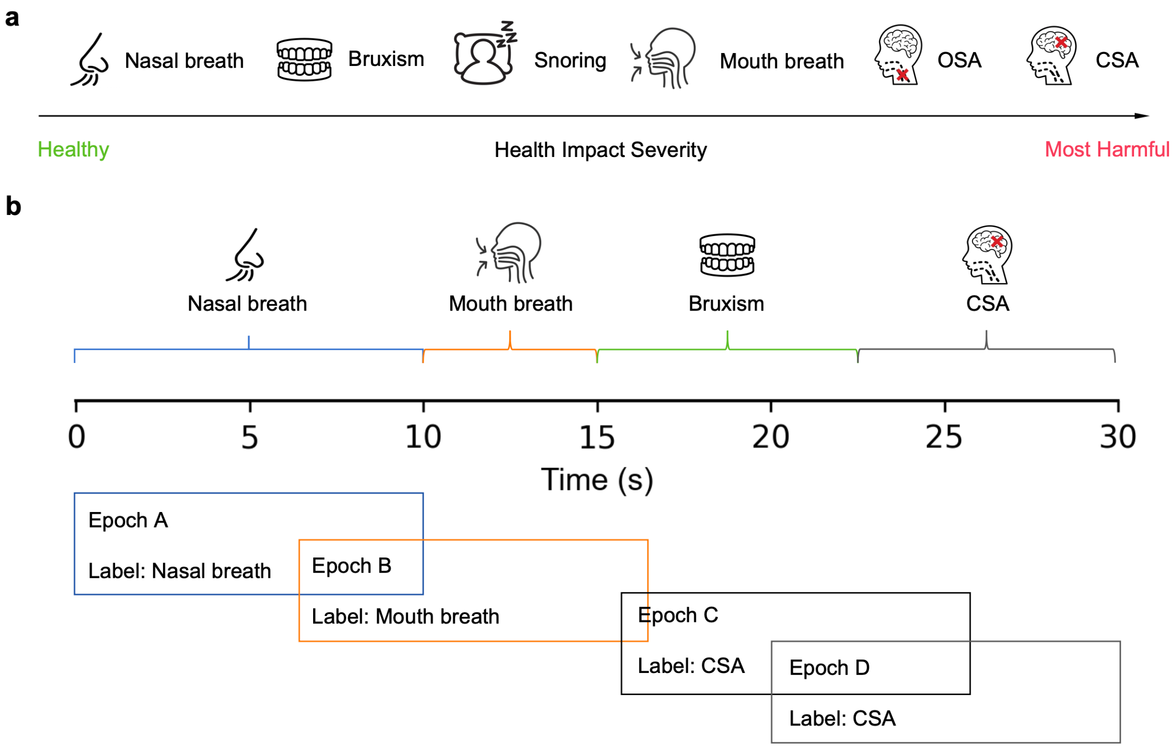

Table S1. Performance comparison with state-of-the-art benchmark neural network backbones.

Participant	Gender	Note	Age	BMI	Experiment Participation
Participant 1	Male	Infrequent snoring, habitual mouth breathing	24	25.3	Main Experiment
Participant 2	Male	Infrequent snoring, habitual nasal breathing	25	19.7	Main Experiment
Participant 3	Female	Frequent snoring, habitual nasal breathing	27	22.1	Main Experiment
Participant 4	Female	Infrequent snoring, habitual mouth breathing	23	23.3	Main Experiment
Participant 5	Male	Frequent snoring, habitual nasal breathing	29	30.8	Main Experiment
Participant 6	Female	Infrequent snoring, habitual mouth breathing	24	18.9	Generalization Experiment
Participant 7	Male	Frequent snoring, habitual nasal breathing	24	26.5	Generalization Experiment

Note: BMI (Body Mass Index) is the weight divided by the square of height (kg/m^2).

Table S2. Performance comparison with state-of-the-art benchmark neural network backbones.

**Backbones**	**Accuracy**	**FLOPS Per Inference**	**Number of Parameters**
1D AlexNet	87.1%	0.12G	24M
1D VGG-16	90.8%	0.60G	36M
1D ResNet-18	93.3%	0.38G	4M
LSTM	91.6%	0.54G	0.8M
GRU	92.3%	0.43G	0.6M
Transformer	96.2%	1.4G	4M
**SleepNet**	98.4%	0.59G	9M
**Pruned SleepNet**	98.6%	0.41G	5M

Table S3. Optimal hyperparameters for the Pruned SleepNet.

	**Optimal hyperparameters of the baseline model**
Optimizer	Ranger
Beta1	0.9
Beta2	0.99
Weight decay	1e-5
Epsilon	1e-8
Learning rate	0.001
Epochs	60
Batch size	32

Movie S1 (separate file). A video summary of the main findings and paper.

SI References

1. D. Pevernagie, R. M. Aarts, & M. D. Meyer, The acoustics of snoring. *Sleep Medicine Reviews* **14**, 131–144 (2010).
2. D. Manfredini, J. Ahlberg, E. Winocur, & F. Lobbezoo, Management of sleep bruxism in adults: a qualitative systematic literature review. *Journal of Oral Rehabilitation* **42**, 862–874 (2015).
3. A. Malhotra *et al.* Metrics of sleep apnea severity: beyond the apnea-hypopnea index. *Sleep* **44**, zsab030 (2021).
4. S. K. Bussadori, L. J. Motta, A. C. R. T. Horliana, E. M. Santos, & A. L. C. Martimbianco, The Current Trend in Management of Bruxism and Chronic Pain: An Overview of Systematic Reviews. *Journal of Pain Research* **13**, 2413–2421 (2020).
5. G. Parati, C. Lombardi, F. Castagna *et al.* Heart failure and sleep disorders. *Nat Rev Cardiol* **13**, 389–403 (2016).
6. S. W. H. Lee, K. Y. Ng & W. K. Chin, The impact of sleep amount and sleep quality on glycemic control in type 2 diabetes: A systematic review and meta-analysis. *Sleep Medicine Reviews* **31**, 91–101 (2017).
7. R. B. Berry *et al.* Rules for scoring respiratory events in sleep: update of the 2007 AASM manual for the scoring of sleep and associated events: deliberations of the sleep apnea definitions task force of the American Academy of Sleep Medicine. *Journal of clinical sleep medicine* **8**, 597-619 (2012).
8. T. Gaisl *et al.* Simulated Obstructive Sleep Apnea Increases P-Wave Duration and P-Wave Dispersion. *PLoS ONE* **11**, e0152994 (2016).
9. D. Aindow, M. Butcher, Films or fabrics: is it time to re-appraise postoperative dressings?. *British journal of nursing* **14**, S15-S20 (2005).
10. C. Ma *et al.* High sensitivity, broad working range, comfortable, and biofriendly wearable strain sensor for electronic skin. *Advanced Materials Technologies* **7**, 2200106 (2022).
11. H. Tu *et al.* A Step Forward for Smart Clothes: Printed Fabric-Based Hybrid Electronics for Wearable Health Monitoring. *Sensors* **24**, 6991 (2024).
12. V. Pecunia *et al.* Roadmap on printable electronic materials for next-generation sensors. *Nano Futures* **8**, 032001 (2024).
13. M. R. Islam *et al.* Advances in Printed Electronic Textiles. *Advanced Science* **11**, 2304140 (2024).
